# mTORC1 and PKB/Akt control the muscle response to denervation by regulating autophagy and HDAC4

**DOI:** 10.1038/s41467-019-11227-4

**Published:** 2019-07-18

**Authors:** Perrine Castets, Nathalie Rion, Marine Théodore, Denis Falcetta, Shuo Lin, Markus Reischl, Franziska Wild, Laurent Guérard, Christopher Eickhorst, Marielle Brockhoff, Maitea Guridi, Chikwendu Ibebunjo, Joseph Cruz, Michael Sinnreich, Rüdiger Rudolf, David J. Glass, Markus A. Rüegg

**Affiliations:** 1 Biozentrum, University of Basel, Klingelbergstrasse 50/70, CH-4056 Basel, Switzerland; 2 Neuromuscular Center, Departments of Neurology and Biomedicine, University Hospital Basel, Klingelbergstrasse 50/70, CH-4056 Basel, Switzerland; 3 Institute for Automation and Applied Informatics, Karlsruhe Institute of Technology, 76344 Eggenstein-Leopoldshafen, Germany; 4 Institute of Toxicology and Genetics, Karlsruhe Institute of Technology, 76344 Eggenstein-Leopoldshafen, Germany; 5 Institute of Molecular and Cell Biology, University of Applied Sciences Mannheim, Mannheim, Germany; 6 Interdisciplinary Center for Neurosciences, Heidelberg University, Heidelberg, Germany; 7 Imaging Core Facility, Biozentrum, University of Basel, Klingelbergstrasse 50/70, CH-4056 Basel, Switzerland; 8 Novartis Institutes for Biomedical Research, Cambridge, USA

**Keywords:** Macroautophagy, Skeletal muscle, Somatic system

## Abstract

Loss of innervation of skeletal muscle is a determinant event in several muscle diseases. Although several effectors have been identified, the pathways controlling the integrated muscle response to denervation remain largely unknown. Here, we demonstrate that PKB/Akt and mTORC1 play important roles in regulating muscle homeostasis and maintaining neuromuscular endplates after nerve injury. To allow dynamic changes in autophagy, mTORC1 activation must be tightly balanced following denervation. Acutely activating or inhibiting mTORC1 impairs autophagy regulation and alters homeostasis in denervated muscle. Importantly, PKB/Akt inhibition, conferred by sustained mTORC1 activation, abrogates denervation-induced synaptic remodeling and causes neuromuscular endplate degeneration. We establish that PKB/Akt activation promotes the nuclear import of HDAC4 and is thereby required for epigenetic changes and synaptic gene up-regulation upon denervation. Hence, our study unveils yet-unknown functions of PKB/Akt-mTORC1 signaling in the muscle response to nerve injury, with important implications for neuromuscular integrity in various pathological conditions.

## Abstract (web summary)

Denervation leads to muscle atrophy and neuromuscular endplate remodeling. Here, the authors show that a balanced activation of mTORC1 contributes to the dynamic regulation of autophagic flux in denervated muscle and that activation of PKB/Akt promotes the nuclear import of HDAC4, which is essential for endplate maintenance upon nerve injury

## Introduction

Skeletal muscle is a highly plastic tissue, whose function strictly depends on neural activity. Nerve injury leads to muscle atrophy and to the remodeling of neuromuscular junctions (NMJs) and non-synaptic muscle regions^1–3^. The mechanisms underlying this integrated muscle response remain poorly understood.

Denervation-induced muscle wasting involves the increased activity of the ubiquitin/proteasome system, with an up-regulation of atrogenes (*e.g. Fbxo32* and *Trim63*) under the control of class II histone deacetylase 4 (HDAC4) and forkhead box O (FoxO) transcription factors^4–8^. FoxO activation is thought to be a consequence of mTORC1- (mammalian Target of Rapamycin Complex 1) induced inhibition of protein kinase B (PKB/Akt), suggesting that mTORC1 activation promotes muscle wasting upon denervation^6^. However, one report rather suggests that mTORC1 activation limits denervation-induced muscle atrophy, by promoting protein synthesis and inhibiting autophagy^9^. Others suggested that both autophagy^5,10,11^ and PKB/Akt^12–15^ are induced following denervation. Thus, the state and the role(s) of PKB/Akt-mTORC1 signaling and autophagy after nerve injury remain largely unknown.

In innervated muscle, acetylcholine receptors (AChRs) and other synaptic proteins are selectively expressed and aggregate at the NMJ. Upon denervation, AChRs are destabilized and their synthesis increases, resulting in a strong increase in their turnover rates^16–22^. In non-synaptic muscle regions, release of the repression of synaptic genes promotes ectopic AChR cluster formation^23–26^. HDAC4 induction and HDAC9 repression control the underlying epigenetic and transcriptional changes following denervation^26–28^. HDAC4 directly represses specific genes (*e.g. Pfkm, Eno3*) and indirectly induces *Myog* (encoding the myogenic factor myogenin), by repressing the genes encoding the co-repressors Dach2 and HDAC9. In turn, myogenin induces both synaptic genes and atrogenes^8,27–29^. However, the mechanisms regulating HDAC4/9 in response to neural activity are unknown.

Here, we examine the role of mTORC1 and PKB/Akt in the muscle response to denervation, focusing on muscle homeostasis and synaptic changes. We report that mTORC1 activation is tightly balanced upon denervation, thereby allowing the muscle-specific, temporal changes in autophagic flux necessary to maintain muscle homeostasis. Simultaneously, PKB/Akt activation promotes HDAC4 nuclear import, to increase synaptic gene expression and AChR turnover, processes that are essential to maintain neuromuscular endplates after nerve injury.

## Results

### Denervation induces PKB/Akt and mTORC1 pathways in muscle

**Fig. 1:**
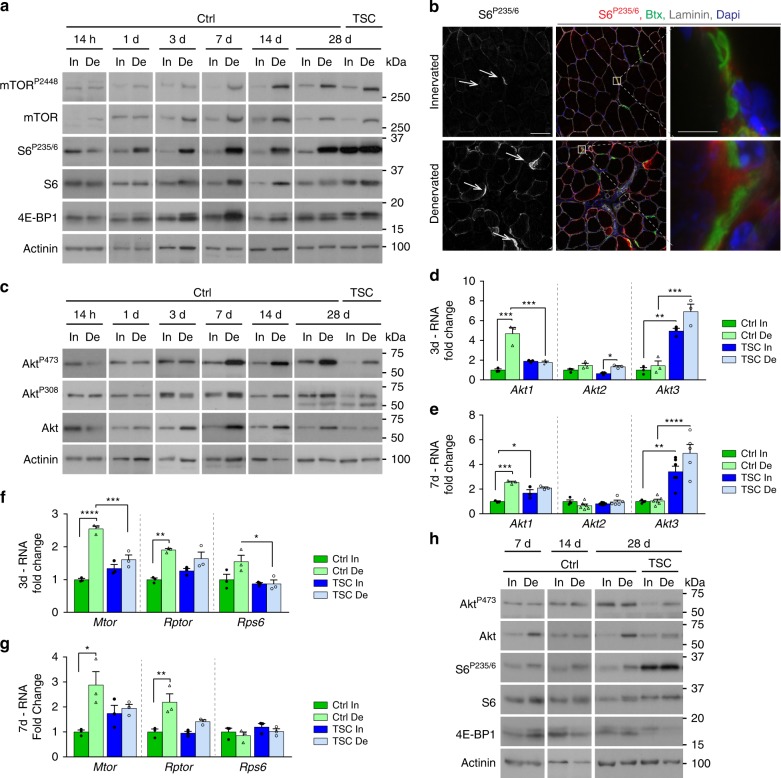
The PKB/Akt-mTORC1 branch is induced upon denervation in TA muscle. **a** Western blot analysis of mTORC1 signaling in control (Ctrl) and TSCmKO (TSC) TA innervated (In) and denervated (De) muscles. Actinin was used as loading control. Representative image of 4 (14 h, 1d) and 3 (3 to 28d) Ctrl and 3 TSCmKO mice. **b** Confocal images of S6^P235/6^ (red), laminin (gray), α-bungarotoxin (green) and Dapi (blue) in TA innervated and denervated muscles (four independent assays). The white arrows point to endplates. Scale bar, 50 µm (5 µm for enlarged view). **c** Western blot analysis of total and phosphorylated PKB/Akt in control and TSCmKO TA innervated and denervated muscles. Actinin was used as loading control. Representative image of 4 (14 h to 3d) and 3 (14 and 28d) Ctrl and 3 TSCmKO mice. **d**–**g** mRNA levels of *Akt1*, *Akt2* and *Akt3* (**d**, **e**) and *Mtor*, *Rptor* and *Rps6* (**f**, **g**) in TA innervated muscle and after 3 (**d**, **f**) and 7 (**e**, **g**) days of denervation, in TSCmKO and control mice. Levels are relative to *Tbp* mRNA and to Ctrl innervated muscle. Values are mean ± s.e.m.; *n* = 4–6 (*Akt2/Akt3*) and 3 (all other genes) mice per genotype; *p < 0.05, **p < 0.01, ***p < 0.001, ****p < 0.0001, two-way ANOVA with a Tukey’s post-hoc analysis. **h** Western blot analysis of PKB/Akt and mTORC1 pathways in *soleus* control and TSCmKO muscles after 7, 14, and 28 days of denervation (*n* = 3 per group). Western blot quantifications are shown in Supplementary Table 1. Source Data are provided in the Source Data File

To determine the state of PKB/Akt-mTORC1 signaling after denervation, we performed Western blot analysis of *tibialis anterior* (TA) muscle at different time points after sciatic nerve cut. After 1 day of denervation, levels of the total and/or phosphorylated forms of mTOR and of the mTORC1 targets, ribosomal protein S6 kinase (p70S6K1), ribosomal protein S6 and 4E-BP1, increased in TA muscle (Fig. 1a and Supplementary Fig. 1a). mTORC1 signaling remained active in denervated muscle throughout the 4 weeks of analysis. Active forms of S6 (S6^P235/6^ and S6^P240^) were enriched near some endplates in innervated muscle and they further accumulated upon denervation (Fig. 1b and Supplementary Fig. 1b, c). Levels of PKB/Akt and of its active, phosphorylated forms (Akt^P473^ and Akt^P308^) were unchanged before 3 days of denervation, but increased thereafter in TA muscle (Fig. 1c). As Tang et al. (2014) previously reported decreased levels of active PKB/Akt^6^, we confirmed the specificity of the phosphorylation at serine 473, by showing that it is lost in denervated muscle from RImKO (*Rictor muscle knockout*) mice, which are depleted of the PKB/Akt kinase mTOR complex 2^30^. In contrast to Akt^P473^, both Akt^P308^ and S6^P235/6^ levels were similar in RImKO and control muscles (Supplementary Fig. 1d). Interestingly, increased protein levels of PKB/Akt correlated with transcriptional up-regulation of *Akt1* at days 3 and 7 post-denervation, while transcript levels of *Akt2* and *Akt3* were unchanged (Fig. 1d, e). Denervation also increased transcript levels of *Mtor* and *Rptor*, but not of *Rps6* (Fig. 1f, g). In TSCmKO (*TSC1 muscle knockout*) mice, which are depleted of the mTORC1 inhibitor TSC1 in skeletal muscle^31,32^, mTORC1 signaling was active in both innervated and denervated muscles (Fig. 1a). Moreover, activation of PKB/Akt was low in the mutant denervated muscle (Fig. 1c), in line with the negative feedback from mTORC1 onto PKB/Akt^31,32^. Notably, up-regulation of *Akt1, Mtor* and *Rptor* was blunted in TSCmKO denervated muscle (Fig. 1d-g), suggesting deregulation of additional upstream mechanisms in mutant muscle. Together, these results point to an early transcriptional induction and a strong activation of both mTORC1 and PKB/Akt in muscle after nerve injury. Of note, denervation caused only minor changes in mTORC1 and PKB/Akt signaling in slow *soleus* muscle of control mice (Fig. 1h), consistent with observations made by others^6^.

### Denervation triggers myopathic alterations in TSCmKO muscle

**Fig. 2:**
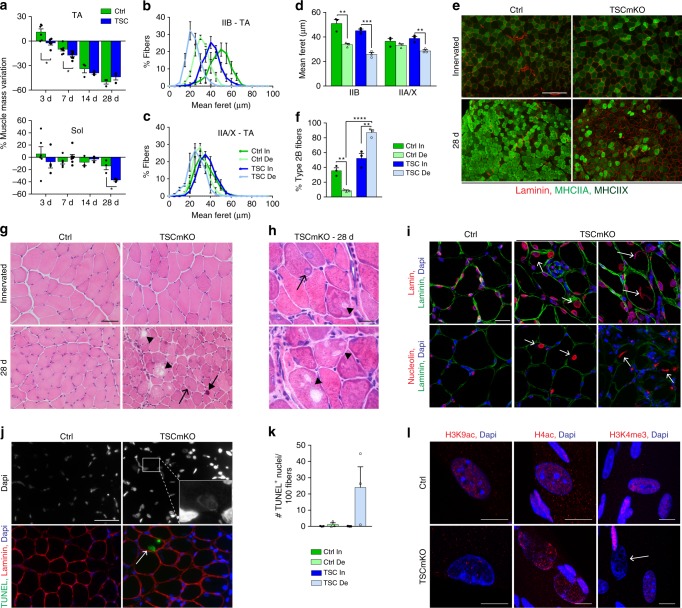
Sustained mTORC1 activation impairs muscle response to denervation. **a** Mass variation for TA and *soleus* muscles in control (Ctrl) and TSCmKO (TSC) mice, after 3, 7, 14 and 28 days of denervation, compared to innervated muscle. *n* = 6 Ctrl, 5/4 TSCmKO (3d TA/Sol), 7/5 (7d TA/Sol) and 3 (14 and 28d) mice per genotype. **b**, **c** Fiber size distribution for type IIB (**b**) and IIA/X (**c**) fibers in TA innervated (In) and denervated (De, 28d) muscles from control and TSCmKO mice. *n* = 3. **d** Minimum mean fiber feret in TA innervated and denervated (28d) muscles from control and TSCmKO mice, distinguishing type IIB and IIA/X fibers. *n* = 3. **e**, **f** Immunostaining of TA control and TSCmKO muscles for MHCIIA/X (green) and laminin (red). Scale bar, 200 µm. Quantification (**f**) gives the proportion of type IIB fibers in TA innervated and denervated (28d) muscles. *n* = 3. **g**, **h** HE coloration of TA control and TSCmKO innervated and denervated (28d) muscles (6 independent muscles per group). Open arrows, arrows and arrowheads point to swollen nuclei, basophilic aggregates and vacuoles, respectively. Scale bar, 50 (**g**) and 10 (**h**) µm. **i** Confocal pictures of lamin (red, top) and nucleolin (red, bottom), laminin (green) and Dapi (blue) in control and TSCmKO denervated (14 and 28d) muscles (3 independent muscles per group). Arrows indicate giant, swollen myonuclei. Scale bar, 20 µm. **j**, **k** TUNEL staining in control and TSCmKO muscles after 28 days of denervation. The arrow points to a positive, swollen myonucleus, as shown in the inset with increased brightness. Scale bar, 50 µm. Quantification in (**k**) gives the number of TUNEL-positive nuclei detected per 100 muscle fibers in control and TSCmKO innervated muscles, and after 28 days of denervation. *n* = 3 mice/group. **l** Confocal pictures of acetylated histones H3 (Lys9; H3K9ac) and H4 (H4ac) and of trimethylated H3 (Lys4, H3K4me3) in denervated (14d) control and TSCmKO muscles (4 independent muscles per group). The arrow indicates a swollen myonucleus. Scale bar, 10 µm. Values are mean ± s.e.m.; two-tailed unpaired Student’s *t-*test (**a**) or two-way ANOVA with Tukey’s post-hoc test (**d**, **f**), *p < 0.05, **p < 0.01, ***p < 0.001. Source Data are provided in the Source Data File

We next investigated whether a sustained activation of mTORC1, by using TSCmKO mice, would affect the muscle response to denervation. Loss of mass in mutant and control mice was similar for TA muscle after 1 month of denervation, but exacerbated in the *soleus* muscle from TSCmKO mice, as compared to control (Fig. 2a). Accordingly, the size of type IIB fibers was similarly reduced in control and TSCmKO muscles upon denervation, while atrophy of type IIA/X and I fibers in TA and *soleus* muscles was stronger in TSCmKO mice than in controls (Fig. 2b–d and Supplementary Fig. 2a-c). Notably, the seeming resistance of *soleus* control muscle to denervation-induced atrophy (Fig. 2a) is likely based on changes in non-muscle tissue, as the mean fiber feret was similarly decreased in *soleus* and TA muscles after 28 days of denervation (−19.7 ± 2.1% in TA *vs*. −22.2 ± 5.7% in *soleus*, mean ± s.e.m). In TA control muscle, denervation caused a major shift in the fiber type proportion, from type IIB fibers to slower fibers expressing myosin heavy chains (MHC) IIA/X (Fig. 2e, f), as has been reported by others^28,33^. In contrast, denervation led to a major loss of type IIA/X fibers and a gain in type IIB fibers in TA (Fig. 2e, f), and even more strikingly, in *soleus* (Supplementary Fig. 2d, e) muscles of TSCmKO mice. Denervation also resulted in the appearance of some embryonic MHC (eMHC)-positive fibers in TSCmKO muscle, indicating ongoing degeneration/regeneration (Supplementary Fig. 2f, g).

Most intriguingly, 4 weeks of denervation triggered a severe myopathy in 3-month-old TSCmKO mice, reminiscent to the changes reported for 9–10 months-old TSCmKO mice^32^. Characteristic features of the myopathy were the accumulation of vacuoles, aggregates and abnormal nuclei in muscle fibers (Fig. 2g, h and Supplementary Fig. 2h, i). Lamin and Dapi staining confirmed the presence of swollen nuclei and suggested that some of the large vacuoles derived from nuclear vacuolization (Fig. 2i and Supplementary Fig. 2j). Nucleoli of giant nuclei were also larger or displayed abnormal shape, suggestive of excessive ribosome biogenesis^9,34^ (Fig. 2i and Supplementary Fig. 2j). Moreover, some nuclei were TUNEL-positive, indicating DNA damage (Fig. 2j, k). However, there was no increase in the number of cleaved caspase3-positive cells or in the levels of endonuclease G^35^ (Supplementary Fig. 2k-m), suggesting that TUNEL-positive nuclei are not dependent on these apoptotic pathways. Expression of *Gadd45*, which has been associated with myonuclear remodeling^36^, was slightly higher in innervated TSCmKO muscle, compared to controls. However, Gadd45 levels were similarly increased in mutant and control muscles after denervation (Supplementary Fig. 2n, o), suggesting that Gadd45 is not responsible for the nuclear changes in TSCmKO muscle. Lastly, immunostaining against modified histones (acetylated H3/H4 and methylated H3K4) was weak or even absent in vacuolated nuclei (Fig. 2l), suggesting that aberrant permissive epigenetic changes are also not causative to the nuclear swelling. Altogether, these results indicate that sustained mTORC1 activation alters the muscle response to denervation, and they show that denervation precipitates the deleterious effects of sustained mTORC1 activation on muscle homeostasis.

### Regulation of autophagy after denervation involves mTORC1

**Fig. 3:**
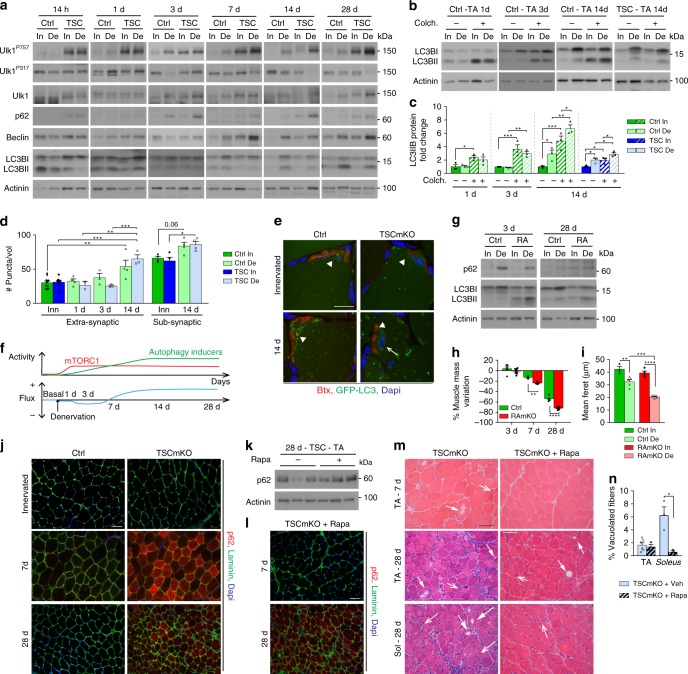
mTORC1 deregulation impairs autophagy dynamics upon denervation. **a–c** Western blot analysis of autophagic markers in TA control (Ctrl) and TSCmKO (TSC) muscles after 14 h, 1, 3, 7, 14 and 28 days of denervation (**a**, representative of 4 (14h-7d) and 3 (14 and 28d) Ctrl and 3 TSCmKO mice per time point), and after 1, 3 and 14 days of denervation coupled with colchicine (colch.) treatment (**b**). Quantification of LC3BII levels in (**b**) is given in (**c**); *n* = 3. **d**, Quantification of GFP-LC3-positive vesicles in TA control and TSCmKO innervated (In) muscles, and after 1, 3 and 14 days of denervation (De), in extra- and sub-synaptic regions. A volume unit (Vol) is 3.2 × 10^3^ µm^3^. *n* = 11, 4, 3, 4 Ctrl and 8, 2, 3, 3 TSCmKO (In, 1, 3 and 14d). **e** Fluorescent images (three independent assays) of TA control and TSCmKO innervated and denervated (14d) muscles showing GFP-LC3-positive puncta (green), α-bungarotoxin (Btx, red) and Dapi (blue). Arrowheads point to endplate region; the arrow indicates a swollen myonucleus. Scale bar, 30 µm. **f** Scheme illustrating changes in mTORC1 activity and autophagic flux in TA muscle upon denervation. **g** Western blot analysis of autophagy markers in innervated TA control and RAmKO (RA) muscles and after 3 and 28 days of denervation. *n* = 3 Ctrl; 4 and 3 RAmKO (3 and 28d) mice. **h** Mass variation for TA muscle from control and RAmKO mice after 3, 7 and 28 days of denervation. *n* = 6, 3 and 5 Ctrl, 8, 4 and 6 RAmKO mice at 3, 7 and 28d. **i** Minimum mean fiber feret in TA innervated and denervated (28d) muscles from control and RAmKO mice. *n* = 3/4 (In) and 5 (De) Ctrl/RAmKO mice. **j** Fluorescent images of p62 (red) and laminin (green) for innervated and denervated TA muscles from control and TSCmKO mice. Representative of 3 Ctrl; 4 and 3 TSCmKO mice at 7 and 28 days. Scale bar, 50 µm. **k** Western blot analysis of p62 in TA denervated (28d) muscles from untreated (−) and rapamycin-treated ( + ) TSCmKO mice. Representative of 3 muscles per group. **l** p62 fluorescent images of TA muscle from rapamycin (Rapa)-treated TSCmKO mice after 7 and 28 days of denervation; 3 independent muscles per group. Scale bar, 50 µm. Compare (**l**) with untreated TSCmKO mice (**j**). **m**, **n** HE staining of TA and *soleus* muscles from untreated and rapamycin-treated TSCmKO mice, after 7 and 28 days of denervation (3 independent muscles per group). Open arrows and arrows point to abnormal nuclei and vacuoles, respectively. Scale bar, 50 µm. Quantification in (**n**) gives the proportion of vacuolated fibers at 28 days in TA and *soleus* muscles. *n* = 6 (untreated TA) and 3 (treated TA; *soleus*). Western blot quantifications are shown in Supplementary Table 1. Values are mean ± s.e.m; two-way ANOVA with Fisher’s (**a**) or Turkey’s (**d**, **i**) post-hoc tests, or two-tailed unpaired Student’s *t-*test (**h**, **n**), *p < 0.05, **p < 0.01, ***p < 0.001, ****p < 0.0001. Source Data are provided in the Source Data File

We have previously shown that autophagy blockade by constant activation of mTORC1 is the main cause for the late-onset myopathy in TSCmKO mice^32^. Because of the phenotypic similarities, we wondered whether autophagy impairment would also underlie denervation-induced myopathy in young TSCmKO mice. As there are conflicting reports on denervation-induced autophagy changes^9,37,38^, we first characterized autophagic flux in muscle from control mice at different time points after sciatic nerve cut. Autophagy markers did not change in control muscle within the first day following denervation (Fig. 3a–d and Supplementary Fig. 3a, b). After 3 days of denervation, autophagy induction was slightly reduced in TA control muscle, as shown by increased p62 levels and limited increase in LC3BII/LC3BI ratio when blocking the degradation steps with colchicine, compared to the contralateral, innervated muscle (Fig. 3a–c and Supplementary Table 1). After prolonged denervation (*i.e*. 7, 14, and 28 days), p62 levels returned to innervated muscle levels, and expression of Beclin1, known to be involved in autophagy induction, increased. Simultaneously, there were high LC3BII levels, which further increased with colchicine (Fig. 3a–c). These results indicate that autophagic flux increases at late stages of denervation in control muscle. Consistently, when using GFP-LC3-expressing mice^39^, GFP-LC3-positive puncta accumulated in muscle fibers after 14 days of denervation, especially near the endplates (Fig. 3d, e). Thus, denervation induces dynamic temporal regulation of autophagy in TA muscle, whereby autophagy induction is low at early stages of denervation and strongly increases at later stages (Fig. 3f). Notably, mTORC1-dependent inhibitory phosphorylation of Ulk1 (Ulk1^P757^)^32,40^ increased in TA control muscle after 3 days of denervation, while levels of the active, phosphorylated form Ulk1^P317^ tended to decrease over time (Fig. 3a). Ulk1^P757^ levels remained high after prolonged denervation, *i.e*. when autophagy was induced. These results are consistent with the strong activation of mTORC1 in denervated TA muscle and point to autophagy inducers promoting autophagic flux after prolonged denervation, despite mTORC1-dependent Ulk1 phosphorylation (Fig. 3f).

To confirm the role of mTORC1 in autophagy regulation in denervated TA muscle, we next measured autophagic flux when modulating mTORC1 activity. First, we injected control mice with rapamycin 12 h before and 12 h after denervation (Supplementary Fig. 3c). As expected, rapamycin acutely (*i.e*. at day 1 after denervation), but transiently (effects were lost by 7 days) inhibited mTORC1 activity (Supplementary Fig. 3d). In control mice, rapamycin treatment slightly increased autophagy 1 day after denervation and this effect persisted until day 7 (Supplementary Fig. 3d). We next analyzed RAmKO (*Raptor muscle knockout*) muscle, in which mTORC1 is inactive^30^, and confirmed that denervation did not change the status of mTORC1 and PKB/Akt signaling in the mutant mice (Supplementary Fig. 3e). LC3BII levels were higher in innervated RAmKO muscle, compared to control muscle, as previously shown^32^, and they further increased 3 and 28 days after denervation (Fig. 3g). Denervation also increased the levels of p62 in 3-day-denervated RAmKO muscle, but less than in controls (Fig. 3g). These results show that autophagic flux increases after denervation in RAmKO TA muscle. Interestingly, the increased autophagic flux was associated with a stronger atrophy response than in controls (Fig. 3h, i). Together, these results indicate that mTORC1 limits autophagy induction, especially at early time points of denervation in control TA muscle, and may thereby limit excessive muscle atrophy.

In TSCmKO mice, denervation did not change LC3B from innervated levels during the first week (Fig. 3a). Thereafter, LC3BII increased in denervated muscle of TSCmKO mice compared to the contralateral, innervated muscle (Fig. 3a–c). Consistently, GFP-LC3-positive puncta accumulated in 14-day-denervated TSCmKO muscle, with autophagic vesicles detected near endplate regions and in the vicinity of swollen nuclei (Fig. 3d, e). These data indicate that prolonged denervation can overcome the mTORC1-mediated blockade of autophagy induction, even in TSCmKO mice. However, LC3BII levels remained lower in denervated TSCmKO muscle than in denervated control muscle, especially upon colchicine treatment, indicating that the flux is still restricted in TSCmKO mice (Fig. 3a–c). Consistently, denervation-induced build-up of p62 was stronger in TSCmKO muscle than in controls (Fig. 3j). To distinguish between the effects of early- and late-stage autophagy impairment on the denervation-induced myopathy in TSCmKO mice, we injected rapamycin 12 h before and 12 h after denervation. This transient rapamycin treatment slightly induced autophagy at day 1 after denervation (Supplementary Fig. 3d), and delayed but did not prevent the accumulation of p62 and the appearance of vacuoles after 4 weeks of denervation (Fig. 3k-n). Together, these results show that mTORC1 activation in TA control muscle blocks the effect of autophagy inducers at early time points of denervation, but is not sufficient to counteract autophagy induction at later stages (Fig. 3f). This temporal regulation is essential to prevent accumulation of damage in the muscle tissue.

Interestingly, autophagy regulation strongly differed in *soleus* muscle. There, autophagy induction increased after 1 day of denervation but was reduced thereafter (Supplementary Fig. 3f-i). After 3 and 28 days of denervation, LC3BII levels were also reduced in *soleus* RAmKO muscle, compared to innervated muscle (Supplementary Fig. 3j), indicating mTORC1-independent inhibition of autophagy. In *soleus* muscle from TSCmKO mice, autophagy induction after one-day denervation was prevented as shown by the limited increase in LC3IIB levels and the accumulation of p62 (Supplementary Fig. 3f, k). Importantly, transient rapamycin treatment (*i.e*. 12 h before and after nerve injury) of TSCmKO mice restored autophagy induction 1 day after denervation in *soleus* muscle (Supplementary Fig. 3l). Rapamycin was also sufficient to prevent the occurrence of the myopathy in denervated *soleus* muscle from TSCmKO mice (Fig. 3m, n and Supplementary Fig. 3k, m). Hence, blockade of autophagy induction at early stages after denervation causes damage to accumulate in the *soleus* muscle from TSCmKO mice. Altogether, these data demonstrate that autophagy regulation is dependent on the duration of denervation and the muscle examined, and is essential for maintaining muscle homeostasis following denervation.

### Sustained mTORC1 activation abolishes endplate maintenance

**Fig. 4:**
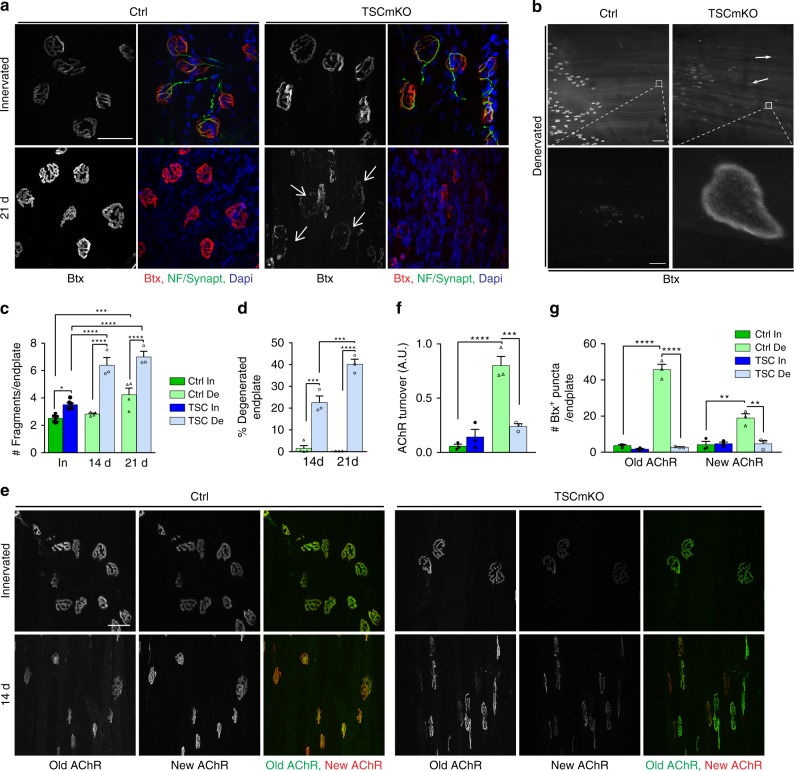
Sustained mTORC1 activation alters endplate maintenance and abrogates AChR turnover. **a** Fluorescent images of TA innervated and denervated (21d), control (Ctrl) and TSCmKO muscles showing NMJ regions stained with α-bungarotoxin (Btx, red) and antibodies against neurofilament and synaptophysin (NF/Synapt, green). Representative of 6 independent muscles per group. Arrows point to degenerated endplates. Scale bar, 50 µm. **b** Fluorescent images of TA denervated control and TSCmKO muscles stained with α-bungarotoxin (Btx). High magnification pictures of the insets (lower panel) show ectopic plaque-like AChR clusters in TSCmKO muscle. Representative of 6 independent muscles per group. Scale bar, 100 (top) and 5 (inset) µm. **c**, **d** Number of fragments per endplate (**c**) and proportion of degenerated endplate (**d**) in TA control and TSCmKO (TSC) muscles after 14 and 21 days of denervation (De). **c**, *n* = 8 (In) and 4 (De) Ctrl, 6 (In) and 3 (De) TSCmKO muscles; **d**, *n* = 4 (Ctrl, 14d) and 3 (all other groups) independent muscles. **e** Fluorescent images of “old” (green) and “new” (red) AChRs in control and TSCmKO muscles (6 TA and 3 EDL muscles per genotype) in innervated muscle and after 2 weeks of denervation. Scale bar, 50 µm. **f**, **g** Quantification of AChR turnover (**f**, EDL muscle) and of endocytosed vesicles containing old or new AChRs (**g**) in innervated (In) and denervated (De) muscles from control and TSCmKO mice. *n* = 3 muscles per group. All values are mean ± s.e.m.; two-way ANOVA with Tukey’s post-hoc test, *p < 0.05, **p < 0.01, ***p < 0.001, ****p < 0.0001. Source Data are provided in the Source Data File

As denervation causes synaptic changes at the neuromuscular endplate and in extra-synaptic regions (*i.e*. >100 µm away from the endplate region)^22,41^, we next compared these changes in TSCmKO and control muscles. Post-synaptic AChRs remained clustered at the endplates and some extra-synaptic AChR clusters appeared in control mice (Fig. 4a, b). In TSCmKO mice, the overall synaptic organization was strongly perturbed in TA and *soleus* muscles, 3 weeks after denervation, as shown by the strong increase in endplate fragmentation, the accumulation of plaque-like AChR clusters throughout the fibers, and the large proportion of degenerated endplates (faintly and dispersedly stained with α-bungarotoxin) (Fig. 4a–d and Supplementary Fig. 4a-c).

To understand these defects, we determined AChR turnover, using established procedures^20^ (see scheme in Supplementary Fig. 4d). As shown by others^16,20,42^, AChR turnover strongly increased in control muscle after denervation (Fig. 4e, f). In striking contrast, old AChRs persisted at the sarcolemma and AChR turnover remained low in denervated TSCmKO muscle (Fig. 4e, f and Supplementary Fig. 4e, f). In parallel, α-bungarotoxin-positive puncta, observed by live imaging, accumulated in control denervated muscle (Fig. 4g). The majority of these AChR-containing puncta represents endocytic vesicles, as they were positive for the markers Rab5/7^43^ (Supplementary Fig. 4g, h). Strikingly, the number of these puncta was strongly reduced in denervated muscle from TSCmKO mice, compared to controls (Fig. 4g and Supplementary Fig. 4g-i). Together, these data reveal that sustained mTORC1 activation hampers the synthesis and internalization of AChRs, and thereby severely alters the maintenance of neuromuscular endplates upon denervation.

### Sustained mTORC1 activation blocks HDAC4 activation

**Fig. 5:**
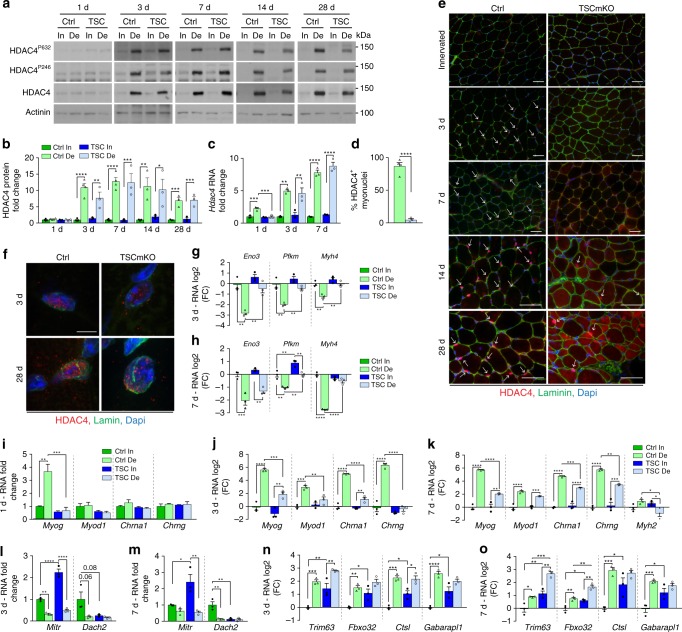
Sustained mTORC1 activation hampers HDAC4 signaling upon denervation. **a**, **b** Western blot analysis of total and phosphorylated HDAC4 in TA control (Ctrl) and TSCmKO (TSC) muscles after 1, 3, 7, 14 and 28 days of denervation (De). Quantification of HDAC4 protein levels is given in **b**; *n* = 4 (1, 3, 7d) and 3 (14, 28d) Ctrl, 3 TSCmKO mice. **c** Transcript levels of *Hdac4* in TA innervated (In) muscle from control and TSCmKO mice, and after 1, 3, and 7 days of denervation. *n* = 3 per group. **d**, **e** Proportion of HDAC4-positive myonuclei in 3-day-denervated control and TSCmKO muscles (**d**), and the corresponding immunostaining showing HDAC4 (red) and laminin (green) in innervated muscle and after 3 to 28 days of denervation (**e**). Representative of 3–4 independent muscles per genotype. Arrows point to HDAC4-positive myonuclei. Scale bar, 50 µm. **d**, *n* = 3 per group. **f** Confocal pictures of endogenous HDAC4 (red) and lamin (green) in myonuclei from control and TSCmKO muscles after 3 and 28 days of denervation (from 4 (3d) and 3 (28d) independent muscles). Scale bar, 5 µm. 3D reconstruction is given in Supplementary Fig. 5a. **g–o** mRNA levels of HDAC4 targets *Eno*, *Pfkm* and *Myh4* (**g**, **h**) of *MyoG*, *Chrna1*, *Chrng, Myod1* and/or *Myh2* (**i**–**k**), of the corepressors *Mitr* and *Dach2* (**l**, **m**), and of *Trim63, Fbxo32, Ctsl* and *Gabarapl1* (**n**, **o**) in TA innervated muscle and after 1 (**i**), 3 (**g**, **j**, **l**, **n**) and 7 (**h**, **k**, **m**, **o**) days of denervation in TSCmKO and control mice. Transcript levels are relative to *Tbp* mRNA and to Ctrl innervated muscle and, analyzed as the log2 fold change (FC) for (**g**, **h**, **j**, **k**, **n**, **o**). *n* = 3 per group. All values are mean ± s.e.m.; two-way ANOVA with Fisher’s (**b**) or Tukey’s (**c**, **g**–**o**) post-hoc tests, or two-tailed unpaired Student’s *t-*test (**d**), *p < 0.05, **p < 0.01, ***p < 0.001, ****p < 0.0001. Western blot quantifications are shown in Supplementary Table 1. Source Data are provided in the Source Data File

Defects observed at the endplates in TSCmKO muscle (*i.e*. low AChR turnover, reduced endocytosed AChRs) are the opposite to those previously reported in Atg7-deficient muscle (*i.e*. increased AChR turnover, accumulation of endocytosed AChRs)^41^, in which autophagy is blocked. These different phenotypes suggest that the endplate defects in TSCmKO muscle are driven by mechanisms independent of autophagy. As an alternative mechanism underlying mTORC1-dependent NMJ perturbation, we examined the HDAC4 signaling, which is involved in the expression of synaptic genes upon denervation^18^. Transcript and protein levels of HDAC4 similarly increased in TA from control and TSCmKO mice after denervation (Fig. 5a-c). However, while HDAC4 accumulated specifically in myonuclei shortly after denervation in control muscle, HDAC4 was undetectable in TSCmKO muscle after 7 days of denervation, and only later accumulated in the cytoplasm and in some giant myonuclei (Fig. 5d, e). Immunoreactivity of HDAC4-positive myonuclei in TSCmKO muscle accumulated at the nuclear envelop rather than in the nucleoplasm (Fig. 5f and Supplementary Fig. 5a). Given the high protein levels detected by Western blot, this suggested that the nuclear import of HDAC4 is impaired in TSCmKO muscle.

To test whether the mislocalization of HDAC4 affected its activity, we quantified the expression of its target genes at 1, 3 and 7 days after denervation. Repression of HDAC4 target genes (*e.g*. muscle specific enolase, *Eno3;* phosphofructokinase, *Pfkm;* MHC type 2b, *Myh4*) was delayed or blunted in denervated TA TSCmKO muscle compared to controls (Fig. 5g, h). Similarly, induction of HDAC4 indirect targets, including genes encoding myogenin (*Myog*), MyoD (*Myod1*), AChR subunits α and γ (*Chrna1/g*), and MHC 2a (*Myh2*), was delayed at day 1 post-injury and/or strongly reduced after 3 and 7 days of denervation in TSCmKO muscle, as compared to controls (Fig. 5i-k). These results are consistent with an impaired function of HDAC4 in mutant muscle. Interestingly, transcript levels of *Mitr*, an alternatively spliced isoform of HDAC9, which represses *Myog* and is a target of HDAC4^28^, were strongly increased in innervated TA TSCmKO muscle (Fig. 5l, m). This may contribute to the altered regulation of myogenic and synaptic genes in mutant muscle. Notwithstanding, *Mitr* levels were efficiently reduced upon denervation in mutant muscle, and expression of the HDAC4 target *Dach2*, which encodes another repressor of *Myog*^28^, was blunted in both innervated and denervated TA TSCmKO muscles (Fig. 5l, m). Of note, similar HDAC4 deregulation was detected in *soleus* muscle from TSCmKO mice (Supplementary Fig. 5b-e).

HDAC4 and FoxO signaling contribute to the up-regulation of atrogenes at 3 and 7 days post-denervation, respectively^6^. As previously shown^31,32^, expression of the atrogenes *Trim63* and *Fbxo32* was high in TSCmKO innervated muscle (Fig. 5n, o). After denervation, the fold change in atrogene transcript levels was reduced in TSCmKO muscle at 3 days (*Trim63*: FC, 3.95 ± 0.41 in Ctrl, 2.74 ± 0.57 in TSCmKO; *Fbxo32*: FC, 2.87 ± 0.35 in Ctrl, 1.90 ± 0.40 in TSCmKO; *n* = 3), but rather increased at 7 days (*Trim63*: FC, 1.94 ± 0.30 in Ctrl, 2.90 ± 0.15 in TSCmKO, p = 0.04; *Fbxo32*: FC, 1.72 ± 0.23 in Ctrl, 2.10 ± 0.22 in TSCmKO; *n* = 3) compared to controls (Fig. 5n, o). This is consistent with a combined, defective HDAC4 function and high FoxO activation in mutant denervated muscle (Supplementary Fig. 5f). Similarly, changes in the expression of autophagy genes were limited in TSCmKO denervated muscle (Fig. 5n, o), which may be related to the HDAC4 defect^44^.

As Ca^2+^/calmodulin-dependent protein kinases II (CaMKIIs) control the trafficking of class II HDACs, we next examined CaMKII activity in TSCmKO muscle. Increase in HDAC4 expression upon denervation was associated with increased levels of the CaMKII-targeted, phosphorylated (Ser247 and 632) forms of HDAC4 in control and TSCmKO muscles (Fig. 5a). Levels of CaMKII isoforms and of their active, auto-phosphorylated forms (Ser287) were unchanged in control muscle after denervation, or even increased at late stages (Supplementary Fig. 5g). Furthermore, the transcript levels of *Camk2d* and *Camk2bM* were unchanged at 3 days of denervation and up-regulated after 7 days (Supplementary Fig. 5h, i). Interestingly, expression of the muscle-specific isoform CaMKIIβM was strongly reduced in TSCmKO muscle, while CaMKIIγ/δ levels were similar to controls (Supplementary Fig. 5g-i). Thus, sustained mTORC1 activation in TSCmKO muscle inhibits the nuclear import and activity of HDAC4, likely independently of CaMKIIs.

### Acute mTORC1 activation impairs endplate remodeling

**Fig. 6:**
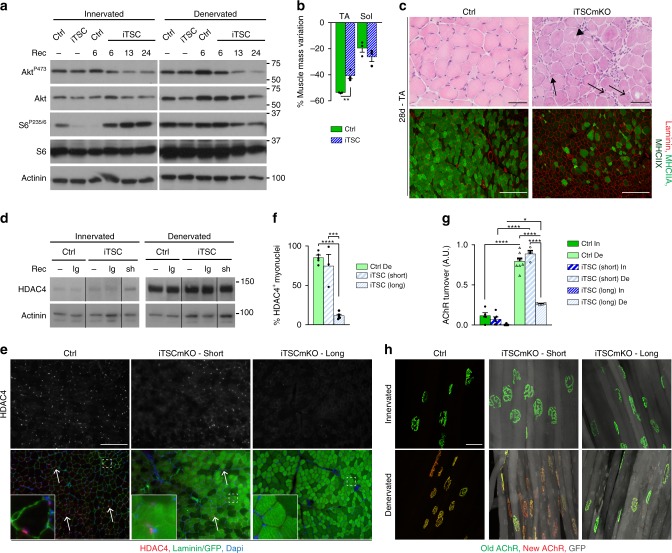
Acute mTORC1 activation impairs HDAC4 function and limits AChR turnover. **a** Western blot analysis of total and phosphorylated levels of PKB/Akt and S6 in TA innervated and 3-day-denervated control (Ctrl) and iTSCmKO (iTSC) muscles, without tamoxifen treatment (−), or 6, 13, and 24 days after recombination induction (Rec). *n* = 3 (untreated, 6 and 13d) and 5 (28d) iTSCmKO mice. **b** Mass variation for TA and *soleus* (Sol) muscles in control and iTSCmKO mice, after 28 days of denervation, as compared to contralateral innervated muscle. *n* = 3 per group. **c** HE coloration (top panel) and fluorescent pictures of MHCIIA/X (bright/dark green) and laminin (red) immunostaining (lower panel) of TA denervated (28d) muscle from control and iTSCmKO mice. Representative of 3 independent muscles per group. Arrows, arrowhead and open arrows point to intracellular aggregates, vacuoles and abnormal giant nuclei, respectively. Scale bar, 50 (top) and 200 (bottom) µm. **d** Western blot analysis of HDAC4 in innervated and denervated (3d) control and iTSCmKO mice after short (sh) or long (lg) recombination induction. **e**, **f** Fluorescent pictures of HDAC4 (red) and laminin / GFP (green) of 3-day-denervated TA muscle from control and iTSCmKO mice with short or long recombination induction. Arrows point to HDAC4-positive myonuclei. Scale bar, 100 µm. Quantification in (**f**) gives the proportion of HDAC4-positive myonuclei. *n* = 5 Ctrl, 3 (short) and 5 (long) iTSCmKO muscles. **g**, **h** AChR turnover quantification for control and iTSCmKO mice with short or long recombination induction. *n* = 4/5/3 (In) and 7/3/3 (De) Ctrl, short and long iTSCmKO muscles. Representative pictures of “old” (green) and “new” (red) AChRs is given in (**h**). GFP appears in gray in iTSCmKO fibers. Scale bar, 50 µm. All values are mean ± s.e.m; two-tailed unpaired Student’s *t-*test (**b**), or one- (**f**) or two- (**g**) way ANOVA with Tukey’s post-hoc test, *p < 0.05, **p < 0.01, ***p < 0.001, ****p < 0.0001. Western blot quantifications are shown in Supplementary Table 1. Source Data are provided in the Source Data File

To test whether HDAC4 deregulation is a consequence of the myopathic changes in TSCmKO mice, we generated a new mouse model (herein called iTSCmKO mice) that allows tamoxifen-induced deletion of *Tsc1* in adult skeletal muscle. One week after tamoxifen-induced recombination, mTORC1 was active, leading to progressive feedback inhibition of PKB/Akt, as previously described in TSCmKO mice^31,32^ (Fig. 6a and Supplementary Fig. 6a). This acute activation of mTORC1 caused some resistance to denervation-induced atrophy in TA but not *soleus* muscle (Fig. 6b). Importantly, after 1 month of denervation, iTSCmKO mice reproduced the alterations observed in TSCmKO muscle (*e.g*. vacuoles, giant nuclei, loss of type IIA/X fibers) (Fig. 6c), with no signs of myopathy in the contralateral, innervated muscle (Supplementary Fig. 6b). As in TSCmKO mice, HDAC4 levels strongly increased upon denervation in iTSCmKO muscle to levels similar to control denervated muscle (Fig. 6d). This response did not depend on whether recombination of floxed *Tsc1* was induced for only 3 days (*i.e*. with limited changes in mTORC1-PKB/Akt activity – referred to as “short”) or more than 1 week (referred to as “long”) before denervation. Notably, despite high protein levels, HDAC4 did not accumulate in myonuclei of denervated iTSCmKO muscle with “long recombination”, but did accumulate after “short recombination” (Fig. 6e, f). Similarly, AChR turnover increased upon denervation in control muscle and iTSCmKO muscle with “short recombination”, but remained low in iTSCmKO muscle with “long recombination” (Fig. 6g, h). These results provide additional evidence that the impaired nuclear import of HDAC4 may be responsible for the endplate defects in TSCmKO mice. They further indicate that these defects are not a consequence of a pre-existing myopathy, but rather the consequence of sustained mTORC1 activation.

### HDAC4 defect contributes to endplate loss in TSCmKO muscle

**Fig. 7:**
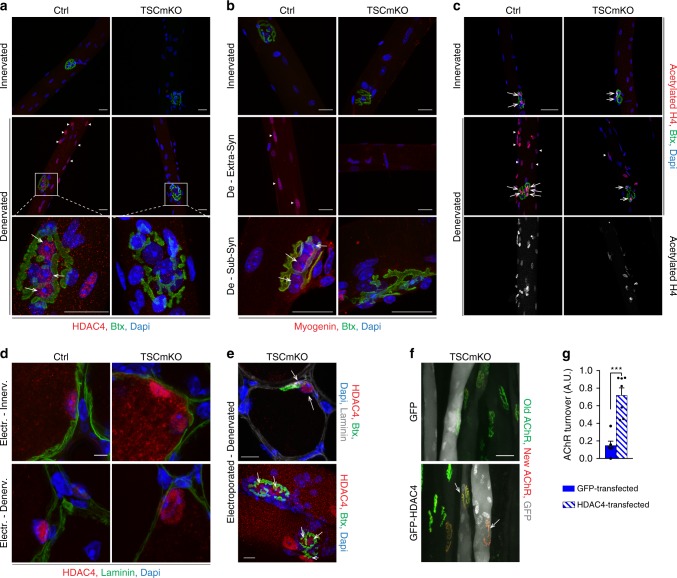
Impaired nuclear import of HDAC4 contributes to endplate defect in TSCmKO muscle. **a**–**c** Confocal pictures of HDAC4 (red, **a**), myogenin (red, **b**) and acetylated histone H4 (red, **c**) combined with α-bungarotoxin (Btx, green) in single fibers isolated from innervated and denervated (De) control (Ctrl) and TSCmKO muscles. Arrows and arrowheads show sub- and extra-synaptic (Syn) myonuclei, respectively. Representative of 4 independent muscles. Scale bar, 25 µm. **d** Confocal pictures of electroporated HDAC4 (red) and laminin (green) in innervated and denervated control and TSCmKO muscles. Representative of 4 independent muscles. Scale bar, 5 µm. 3D reconstruction is given in Supplementary Fig. 7d. **e** Confocal pictures of electroporated HDAC4 (red), α-bungarotoxin (Btx, green) and laminin (gray) on muscle section (upper panel) or in isolated fibers (bottom panel) from TSCmKO denervated muscle. Arrows point to sub-synaptic nuclei. Representative of 4 independent muscles. Scale bar, 10 µm. **f**, **g** Representative confocal pictures of “old” (green) and “new” (red) AChRs in TSCmKO denervated muscles (14d), electroporated with GFP or GFP-HDAC4 (gray). Scale bar, 50 µm. Turnover quantification is given in (**g**); values are mean ± s.e.m.; *n* = 6 GFP and 7 HDAC4 independent assays (number of mice electroporated; 136 and 50 fibers quantified, respectively); two-tailed unpaired Student’s *t-*test, ***p < 0.001. Source Data are provided in the Source Data File

Maintenance of neuromuscular endplates upon denervation involves the up-regulation of synaptic genes in sub- and extra-synaptic regions^45,46^. To confirm the role of HDAC4 in endplate degeneration in TSCmKO mice, we first analyzed HDAC4 localization throughout muscle fibers. While HDAC4 was undetectable in single fibers isolated from innervated muscle, it accumulated strongly in sub- and extra-synaptic myonuclei of control muscle upon denervation (Fig. 7a). Similarly, myogenin was detected in both sub- and extra- synaptic nuclei in denervated control fibers (Fig. 7b). In contrast, HDAC4 and myogenin were detected neither in sub- nor in extra-synaptic myonuclei of denervated TSCmKO muscle (Fig. 7a, b). Similar results were obtained using immunostaining on muscle sections (Supplementary Fig. 7a, b). As HDAC4 has been shown to inhibit HDAC9 and thereby to increase histone acetylation upon denervation^26^, we next analyzed levels of histone acetylation in control and TSCmKO muscles. While acetylation of histone H4 increased upon denervation in both sub- and extra-synaptic regions in control muscle, the signal remained low in TSCmKO denervated fibers (Fig. 7c). A similar difference between control and TSCmKO muscles was observed with acetylated histone H3 (Supplementary Fig. 7c). These results suggest that HDAC4 induction contributes to synaptic gene up-regulation in sub- and extra-synaptic regions, and they show that this activity is abrogated in TSCmKO muscle.

To assess whether HDAC4 deregulation is responsible for the defective endplate maintenance in TSCmKO and iTSCmKO muscles, we next electroporated plasmids encoding GFP-tagged HDAC4 into denervated mutant muscle. Although the nuclear accumulation of the electroporated HDAC4 was less in TSCmKO fibers than in control muscle (Fig. 7d and Supplementary Fig. 7d), HDAC4 overexpression was sufficient to drive some HDAC4 into myonuclei, including sub-synaptic myonuclei, in TSCmKO muscle (Fig. 7e). Thus, we next combined HDAC4 overexpression with the measurement of AChR turnover in denervated TSCmKO muscle. Importantly, AChR turnover was strongly increased in HDAC4-transfected fibers from TSCmKO muscle, but remained low in GFP-electroporated mutant fibers (Fig. 7f, g). These results indicate that defective HDAC4 activity contributes to the loss of neuromuscular endplates upon denervation in TSCmKO muscle.

### PKB/Akt activation promotes HDAC4 and endplate remodeling

**Fig. 8:**
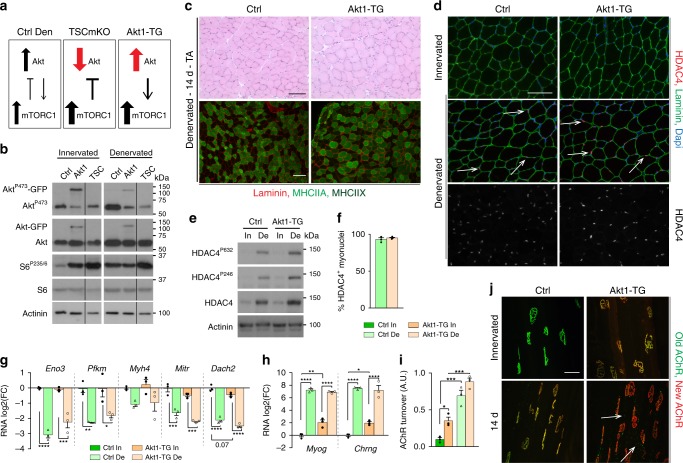
PKB/Akt activation is required for HDAC4 nuclear import and promotes endplate remodeling. **a** Changes in mTORC1 and PKB/Akt pathways in control (Ctrl), TSCmKO and Akt1-TG denervated muscles. **b** Western blot analysis of total and phosphorylated levels of endogenous and transgenic (Akt1-GFP) PKB/Akt, and of S6 in TA innervated and 3-day-denervated control, Akt1-TG (Akt1) and TSCmKO (TSC) muscles. Mice were treated with tamoxifen the day prior denervation and over the experiment period. *n* = 3 Ctrl, 3 Akt1-TG and 2 TSCmKO mice. **c** HE coloration (top panel) and fluorescent pictures of MHCIIA/X (bright/dark green) and laminin (red) immunostaining (bottom panel) of TA denervated (14d) muscle from control and Akt1-TG mice. Representative of 3 Ctrl, 3 Akt1-TG and 4 TSCmKO mice. Scale bar, 100 µm. **d** Immunofluorescent pictures of TA denervated (3d) muscle (6 independent muscles) from control and Akt1-TG mice, showing HDAC4 (red) and laminin (green). Arrows point to HDAC4-positive myonuclei. Scale bar, 100 µm. **e** Western blot analysis of total and phosphorylated HDAC4 in TA innervated (In) and denervated (De; 3d) muscles from control and Akt1-TG mice. *n* = 3 per group. **f** Proportion of HDAC4-positive myonuclei in TA denervated (3d) muscle from control and Akt1-TG mice. *n* = 3 per group. **g**, **h** Transcript levels of *Eno2, Pfkm*, *Myh4, Mitr* and *Dach2* (**g**) and *Myog* and *Chrng* (**h**) in TA innervated and denervated muscles, from control and Akt1-TG mice. Transcript levels were normalized to *Tbp* mRNA and to Ctrl innervated muscle, and analyzed as the log2 of the fold change. *n* = 3 per group. **i**, **j** AChR turnover quantification in control and Akt1-TG innervated muscles, and 14 days after denervation (**i**). *n* = 3 per group. Representative pictures of “old” (green) and “new” (red) AChRs are given in (**j**). Arrows point to internalized AChRs. Scale bar, 50 µm. All values are mean ± s.e.m.; two-way ANOVA with Tukey’s post-hoc test, *p < 0.05, **p < 0.01, ***p < 0.001, ****p < 0.0001. Western blot quantifications are shown in Supplementary Table 1. Source Data are provided in the Source Data File

Since control and TSCmKO muscles both show strong mTORC1 activation upon denervation, but differ in the activation state of PKB/Akt (Fig. 8a), we tested whether PKB/Akt inhibition would be responsible for HDAC4 defects and endplate degeneration in TSCmKO muscle. To test this, we used transgenic mice (Akt1-TG mice) expressing an active, myristoylated form of Akt1, fused with EGFP and ERT2. In the absence of tamoxifen, the fusion protein is immediately degraded, while the binding of tamoxifen to ERT2 renders the protein stable. Thus, injection of tamoxifen leads to the immediate activation of PKB/Akt, which in turn strongly activates mTORC1 (Fig. 8b). Hence, mTORC1 activation is similar in Akt1-TG and TSCmKO muscles, while PKB/Akt is active in Akt1-TG muscle, but inhibited in TSCmKO muscle (Fig. 8a). Activation of Akt1 for 2 weeks was sufficient to increase muscle mass in innervated muscle, and to limit muscle mass loss after denervation (Supplementary Fig. 8a), as previously reported^47^. This effect was related to the resistance to denervation-induced atrophy of type IIA/X fibers in Akt1-TG muscle (Supplementary Fig. 8b, c). After 14 days of denervation, Akt1-TG muscle showed only minor myopathic signs and there was no change in fiber type proportion (Fig. 8c and Supplementary Fig. 8d), in contrast to TSCmKO muscle. As in controls, HDAC4 was undetectable by immunostaining in innervated Akt1-TG muscle (Fig. 8d), despite slightly higher protein levels (Fig. 8e). Importantly, protein levels of HDAC4 strongly increased after denervation in Akt1-TG muscle, and it accumulated in myonuclei, with higher signal intensity compared to control muscle (Fig. 8d–f). Expression of HDAC4 targets was efficiently repressed after denervation in mutant muscle (Fig. 8g). Inversely, levels of *Myog* and *Chrng* were increased in Akt1-TG innervated muscle, compared to control, and reached similarly high levels in control and mutant muscles after denervation (Fig. 8h). Hence, in contrast to the blunted activation of HDAC4 detected in TSCmKO and iTSCmKO muscles, PKB/Akt activation promotes HDAC4 signaling, despite the acute activation of mTORC1.

As HDAC4 defect contributes to endplate degeneration in TSCmKO denervated muscle, we examined synaptic changes in Akt1-TG muscle. Endplate organization in Akt1-TG mice looked similar to controls both in innervated and denervated muscles (Supplementary Fig. 8e-g). Interestingly, AChR turnover was higher in the innervated muscle of Akt1-TG mice than in controls and further increased after denervation (Fig. 8i, j). This indicates that acute mTORC1 activation, when combined with PKB/Akt activation, does not impinge on synaptic changes upon denervation and, that the defects observed in TSCmKO and iTSCmKO muscles are rather due to PKB/Akt inhibition (Fig. 8a).

**Fig. 9:**
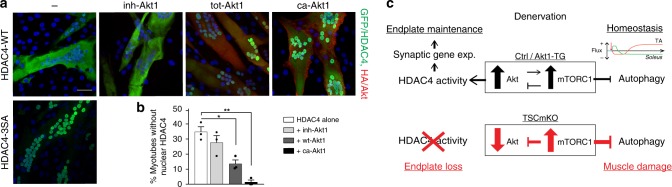
PKB/Akt promotes HDAC4 nuclear import in muscle cells. **a**, **b** Fluorescent pictures of C2C12 myotubes co-electroporated with a wild-type form (WT) of HDAC4 tagged with GFP or with a nuclear mutant (HDAC4-3SA), together with an inhibited (inh), a wild-type (wt) or a constitutively active (ca) form of Akt1 tagged with HA. Scale bar, 50 µm. The quantification in (**b**) gives the proportion of myotubes with only cytoplasmic HDAC4 localization; data are mean ± s.e.m.; total myotubes counted = 406 (−), 322 (inh), 356 (wt), 205 (ca); one-way ANOVA with Tukey’s post-hoc test, *p < 0.05, **p < 0.01. **c** Scheme illustrating the role of mTORC1 and PKB/Akt in the muscle response to denervation. Source Data are provided in the Source Data File

To confirm the role of PKB/Akt in HDAC4 regulation, we lastly co-electroporated C2C12 myotubes with plasmids encoding the wild-type form or a nuclear mutant (HDAC4-3SA) of GFP-tagged HDAC4^27^, together with plasmids coding for either a wild-type, a myristoylated (*i.e*. active) or an inhibited form of HA-tagged Akt1^48,49^. Electroporation was done at 6 days of differentiation to avoid any effect on cell fusion and growth. Wild-type HDAC4 localized in cytoplasm and/or nuclei, while HDAC4-3SA was only detected in nuclei (Fig. 9a). Co-electroporation of wild-type HDAC4 with the inhibited form of Akt1 did not modify the subcellular localization of HDAC4, compared to cells electroporated with HDAC4 alone (Fig. 9a, b). In contrast, the proportion of myotubes with only cytoplasmic HDAC4 was decreased when they were co-transfected with wild-type Akt1, and even more so with the myristoylated form of Akt1 (Fig. 9a, b). This shows that activation of PKB/Akt promotes the nuclear import of HDAC4 in myotubes. Altogether, these results indicate that PKB/Akt activation is essential for the nuclear import and the activity of HDAC4 in muscle after denervation, and thereby for the maintenance and remodeling of neuromuscular endplates (Fig. 9c).

## Discussion

Nerve injury leads to major changes in skeletal muscle, including remodeling of the post-synaptic apparatus and loss of muscle mass. The molecular mechanisms responsible for these changes remain largely unknown. We here establish that mTORC1 and PKB/Akt are activated upon denervation, and that a tight regulation of their activity is required to maintain muscle homeostasis. Sustained or acute mTORC1 activation leads to severe muscle alterations after nerve injury, related to autophagy impairment, and abrogates physiological muscle responses to denervation (*i.e*. fiber type switch, gene up-regulation, increased AChR turnover). We show that this effect is caused by inhibition of PKB/Akt, which abrogates the nuclear import of HDAC4 and, thereby synaptic gene up-regulation in the denervated muscle.

Previous reports suggested that denervation activates mTORC1, although its role in denervation-induced atrophy remains debated^6,9^. Similarly, some studies pointed to an activation of PKB/Akt upon denervation, while Tang et al. reported that the signaling is inhibited^6,12–15^. We now establish that denervation triggers activation of both mTORC1 and PKB/Akt, accompanied by a transcriptional up-regulation of the *Akt1*, *Mtor*, and *Rptor* genes. We further demonstrate that to maintain homeostasis, mTORC1 activation must be tightly controlled in the denervated muscle. This effect is dependent on the dynamic regulation of autophagic flux upon denervation. In particular, in TA muscle, mTORC1 activation inhibits autophagy at early stages, and may thereby limit excessive muscle atrophy. In contrast, at late stages, autophagy induction increases despite mTORC1 activation and the subsequent inhibition of Ulk1, which likely involves alternative pathways triggering autophagy induction^50^. In *soleus* muscle, autophagy is induced shortly after denervation and reduced later independent of mTORC1. Hence, autophagy re-induction at late stages may be an adaptive mechanism to cope with the increase in protein synthesis related to mTORC1 activation detected in TA, but not *soleus*, muscle. Constant activation of mTORC1 by genetic manipulation restricts autophagy in TA and *soleus* denervated muscles (especially at late and early time points, respectively), and leads to an accumulation of autophagy-related alterations. Inversely, mTORC1 inactivation increases autophagic flux in denervated TA muscle, which correlates with an exacerbated muscle atrophy.

Importantly, besides their role in muscle homeostasis, we unveil a determinant, yet-unknown function of mTORC1 and PKB/Akt in muscle physiology. Although mTORC1 becomes activated in control muscle after denervation, constant activation of mTORC1 with a consecutive inhibition of PKB/Akt (TSCmKO and iTSCmKO mice) abrogates several hallmarks of denervation. In this case, HDAC4 nuclear accumulation was hampered, while its protein levels efficiently increased. Several kinases have been shown to modulate HDAC4 nuclear import, such as CaMKIIs^51,52^ and PKA/C^53–55^. We now show that activation of PKB/Akt is sufficient to drive HDAC4 into myonuclei in cultured myotubes, and is required for HDAC4 nuclear accumulation in denervated muscle. The mislocalization of HDAC4, and the subsequent deregulation of its target genes, are likely responsible for several defects observed in TSCmKO and iTSCmKO denervated muscles. In particular, the abnormal fiber type switch in denervated TSCmKO muscle correlates with the abnormal regulation of *Myh4* and *Myh2*, two targets of HDAC4. Similarly, recent studies suggested that the main driver for AChR destabilization after nerve injury is the incorporation of new AChRs at the membrane^18^. Although not yet clearly established, it is likely that the up-regulation of synaptic genes in both sub- and extra-synaptic regions supports the increased turnover of synaptic proteins at the neuromuscular endplate, and thereby its maintenance. Consistently, we show that HDAC4 is detected in both sub- and extra-synaptic myonuclei upon denervation. Moreover, together with the defective nuclear import of HDAC4, the induction of myogenic and synaptic genes and the increase in AChR turnover were hampered in TSCmKO muscle. HDAC4 overexpression was sufficient to drive HDAC4 nuclear accumulation in sub- and extra-synaptic myonuclei, and to restore AChR turnover in TSCmKO muscle. Up-regulation of synaptic genes and increased AChR turnover in innervated Akt1-TG muscle further support the role of PKB/Akt in the regulation of HDAC4, although one cannot rule out the contribution of HDAC4-independent mechanisms in the effects observed. Based on our results and consistent with previous reports^27,28,56^, it is also likely that HDAC4-independent mechanisms contribute to *Mitr/Dach2* repression in muscle and, that alternative effectors mediate the effect of HDAC4 on synaptic gene regulation upon denervation.

In conclusion, our work has unraveled important roles of the mTORC1 and PKB/Akt pathways in the muscle response to denervation, which includes the control of muscle homeostasis, as well as the maintenance of neuromuscular endplates. PKB/Akt-dependent regulation of HDAC4 is an important contributor of this response. Hence, one should consider the deregulation of the PKB/Akt-mTORC1 axis as a potent factor in the loss of neuromuscular integrity in neuromuscular diseases and systemic pathological conditions, such as aging.

## Methods

### Animals

Generation and genotyping of RAmKO, TSCmKO, and GFP-LC3 transgenic mice were described previously^30,39^. Control mice for RAmKO and TSCmKO mice were littermates that were floxed for *Rptor* (gene encoding raptor) or *Tsc1*, but did not express Cre-recombinase. Inducible TSCmKO mice (iTSCmKO) were obtained by breeding *Tsc1*-floxed mice with mice expressing the Cre recombinase in skeletal muscle upon tamoxifen injection (inducible modified *HSA* promoter, *HSA*-MerCreMer^57^). Recombination was induced by 5 tamoxifen injections, and denervation was performed from 3 (short) to 10–21 (long) days after recombination induction. Control mice for iTSCmKO mice were littermates that were floxed for *Tsc1*, treated with tamoxifen, but did not express Cre-recombinase. Inducible Akt1-TG mice were developed by recombinase mediated cassette exchange into the ROSA 26 locus using a modified embryonic stem cell line. A neomycin-resistant inducible Akt1 cassette was cloned between lox 511 and lox P sites, which allowed cassette exchange in the presence of Cre. After cassette exchange, neomycin-resistant cells were used for microinjection into blastocysts and eventual implantation into surrogate females to generate the inducible Akt1-TG line. Akt1-TG mice were pre-treated with tamoxifen the day before denervation, and were then treated over the period of experiment. Controls for Akt1-TG mice were littermate wild-type mice treated with tamoxifen. Sciatic nerve cut and in vivo muscle electroporation were conducted as described previously^31,58^. Upon denervation, muscle mass variation (%) was calculated as the difference of the mass of the denervated and innervated muscles (from the contralateral leg), normalized to the mass of the innervated muscle. In some experiments, mice were intra-peritoneally injected with colchicine (Sigma, 0.4 mg/kg) or rapamycin (LC Laboratories, 1.5 mg/kg)^47,59^. Mice were maintained in a conventional facility with a fixed light cycle (23 °C–12 h dark-light cycle). All animal studies were performed in accordance with the European Union guidelines for animal care and approved by the Swiss authorities.

### Cell culture

C2C12 cells were obtained from ATCC (CRL-1772). Myoblasts were grown in Dulbecco’s modified Eagle’s medium (DMEM; Sigma, D5796) supplemented with 20% fetal bovine serum and 1% penicillin-streptomycin (pen/strep). They were differentiated into myotubes by switching to differentiation medium (DMEM, 2% horse serum, 1% pen/strep). Electroporation of myotubes was done after 6 days in differentiation medium, using NEPA21 electroporator (NEPAgene) with the CUY900-13-3-5 Cell-Culture-Plate Electrode, in 24-well plate. Cells were fixed, 24 hr after electroporation, with 2% paraformaldehyde (PFA), 2% sucrose, washed with PBS (pH 7.4) and 0.1 M glycine, and analyzed by immunostaining.

### Transcript expression analyses

Total RNA was extracted with the RNeasy Fibrous Tissue Mini Kit (Qiagen). Quantitative PCR was performed on DNAse-treated RNA, reverse transcribed to cDNA using the SuperScript III First-Strand Synthesis System (Invitrogen), amplified with the Applied Biosystem Power Sybr Green Master Mix. Data were analyzed using StepOne software and normalized to *Tbp* expression. Primers are listed in Supplementary Table 2.

### Antibodies

All primary antibodies were used at 1/1000 for Western blot; when the antibody was used for IHC, the dilution is indicated in the list. The following antibodies were used: PKB/Akt (#9272), Phospho-Akt^Ser473^ (#9271), Phospho-Akt^Ser308^ (#9275), p70 S6 kinase (#9202), Phospho-p70 S6 kinase^Thr389^ (#9205), S6 Ribosomal Protein (#2217), Phospho-S6 Ribosomal Protein^Ser235/6^ (#2211; 1/100 for IHC), Phospho-S6 Ribosomal Protein^Ser240^ (#2215; 1/100 for IHC), LC3B (#2775), Ulk1 (#8054), Phospho-Ulk1^Ser757^ (#6888), Phospho-Ulk1^Ser317^ (#6887) Beclin1 (#3495), HDAC4 (#15164 and #7628; 1/5000 for IHC), Phospho-HDAC4^Ser246^ (#3443), Phospho-HDAC4^Ser632^ (#3424), nucleolin (#14574; 1/500 for IHC), endonuclease G (#4969), Gadd45 (#4632), Rab5 (#2143; 1/100 for IHC), Rab7 (#9367; 1/100 for IHC) from Cell Signaling; α-actinin (A5044) and Neurofilament 200 (N4142; 1/2000 for IHC) from Sigma; p62 (GP62C; 1/300 for IHC) from Progen; myogenin (F5D; 1/100 for IHC), Myosin Heavy Chain types I (A4.840; 1/300 for IHC), IIA/IIX (A4.74; 1/300 for IHC), IIB (BF-F3; 1/300 for IHC) from the *Developmental Studies Hybridoma Bank*; Laminin (ab11575 and ab11576; 1/500 for IHC) from Abcam; Lamin B from Santa Cruz (C-20); Synaptophysin (A0010; 1/200 for IHC) from Dako; acetylated Histones H3 (17–658; 1/1000 for IHC) and H4 (06–598; 1/1000 for IHC), Trimethyl Histone H3 (Lys4 - 17–614; 1/500 for IHC) from Millipore Merck.

### Western blotting

TA and *soleus* muscles were frozen and powdered in liquid nitrogen. They were lysed in RIPA buffer (50 mM Tris HCl pH8, 150 mM NaCl, 1% NP-40, 0.5% sodium deoxycholate, 0.1% SDS, 1% Triton-X, 10% glycerol) with protease and phosphatase inhibitor cocktail tablets (Roche). Cell lysates were incubated on ice for 2 h, sonicated two times for 10 s and centrifuged at 10,000 × *g* for 20 min at 4 °C. Cleared lysates were used to determine total protein amount (BCA Protein Assay, Pierce). Proteins were separated in 7 or 15% polyacrylamide SDS gels and transferred to nitrocellulose membrane.

### Histology analyses

Muscles were dissected and frozen in liquid nitrogen-cooled isopentane; eight µm muscle cryosections were used for histology analyses. Cryostat sections were stained with Hematoxylin/Eosin (HE) according to classical methods^60^. Light microscopy was performed using an upright microscope (Leica and Olympus), and pictures were captured using a monochrome camera (DS-Ri1, Nikon).

For MHC staining, sections were fixed with 100% methanol, followed by microwave oven antigen retrieval in citric acid. For HDAC4 and FoxO3 staining, sections were fixed with PFA 4%. Sections were then blocked with 3% IgG free bovine serum albumin and AffiniPure Mouse IgG Fab Fragments (Jackson ImmunoResearch). They were incubated sequentially with primary and secondary fluorescent antibodies (Invitrogen) and mounted in Vectashield DAPI (Vector). TUNEL staining was done with the In Situ Cell Death Detection Kit, Fluorescein (Roche). Images were captured using the analySIS software (Soft Imaging System) with a fluorescent Leica microscope. Fiber size distribution was determined based on laminin immunostaining (analySIS software).

For GFP-LC3 detection, mice were perfused with 4% PFA in PBS. TA and *soleus* muscles were excised, fixed in the same fixative and treated with 30% sucrose in PBS overnight. Cryosections were stained with α-bungarotoxin-Alexa555 (Invitrogen), washed with PBS and mounted. Images were recorded using a Leica confocal microscope with 63x objective. The number of GFP-LC3 puncta was counted on 3D reconstructed images with the Imaris software. Six image stacks in extra-synaptic regions, and 15 sub-synaptic fields were quantified for each muscle. The average numbers of GFP-LC3 puncta per volume unit (20.46 × 20.46 × 7.55 µm^3^) were used for statistical analyses. All GFP quantifications were done in a double-blind way.

### Staining of muscle bundles and single isolated fibers

To analyze NMJ organization, muscles were bathed *ex vivo* (2 µg/ml) with α-bungarotoxin-Alexa555 (Invitrogen) for 30 min, before being washed and fixed with 4% PFA. Muscle bundles were cut, permeabilized in PBS, 1% Triton-X100, and blocked in PBS, 1% BSA, 0.1% Triton-X100. Bundles were then successively incubated with primary antibodies against Neurofilament and Synaptophysin (to stain pre-synaptic compartment), and the corresponding secondary antibodies (Invitrogen). When combined with electroporation, mice were perfused with 4% PFA in PBS, and muscle were post-fixed for 15 min, before processing muscle bundles. Endplates were considered as degenerated when they showed no obvious dense AChR-positive dense fragment, or when more than 30 fragments were observed. Immunostaining on single fibers was done on fibers isolated from PFA-fixed muscle and permeabilized with PBS, 3% BSA, 0.5% Triton^26^. Primary antibodies were incubated overnight, followed by over day washing, and incubation with the corresponding secondary antibodies (Invitrogen) and fluorescent α-bungarotoxin to distinguish extra- and sub-synaptic regions. More than 30 fibers were used per analysis. Images were recorded using a Leica confocal microscope with ×40 to ×100 objectives.

### AChR turnover

AChR turnover was assessed by injecting α-bungarotoxin-Alexa647 and -Alexa555 (25 pmoles - Invitrogen) into TA/EDL muscles at days 1 and 10, respectively (5 and 14 days after nerve cut when combined with denervation). Analysis was conducted in living muscle as previously done^20,41^ or with muscle bundles. Analysis of fixed whole-mount preparations of TA and EDL muscle fiber bundles gave similar results to live imaging of muscle (see Supplementary Fig. 4e, f). For turnover quantification, images were recorded using a Leica confocal microscope with ×63 objective. Pixel dominance (old or new receptors) was calculated using Fiji and Matlab software^20,41^.

### Images and statistical analyses

Images were analyzed with the analySIS (Soft Imaging System), Imaris, Fiji and Matlab software. Results are expressed as mean ± SEM of independent animals, with *n* (number of individual experiments) ≥3. Statistical comparison was performed using two-tailed Student’s *t*-test or one/two-way ANOVA test, with prior log transformation of the data, dependent on the conditions. A 0.05 level of confidence was accepted for statistical significance. All *p* values are provided in the Source Data File^27,48,49^.

### Reporting Summary

Further information on research design is available in the Nature Research Reporting Summary linked to this article.

## Supplementary information

Supplementary information

Reporting Summary

Source Data

## Data Availability

The data presented in this study are available from the corresponding authors upon reasonable request. The source data underlying Figs. 1a,c–h, 2a-d,f,k, 3a-d, g-i, k, n, 4c, d, f, g, 5a–d, g–o, 6a, b, d, f, g, 7g, 8b, e–i, 9b, and Supplementary Figs 1a,d, 2a-d, g, l-o, 3a, d-h, j, l, m, 4b, c, e, f, h, i, 5b, d, e, g-i, 8a-d, f, g are provided as a Source Data file.

## Code availability

The code used for AChR turnover analysis has been described previously^20,41^, and is available from the authors upon reasonable request.
