# Supplementary material for: mTORC1 and PKB/Akt control the muscle response to denervation by regulating autophagy and HDAC4
Source: Nat Commun. 2019 Jul 18;10:3187. doi: 10.1038/s41467-019-11227-4 (PMC6639401; doi:10.1038/s41467-019-11227-4)
Supplement: Supplementary file 1 — Supplementary information [file 41467_2019_11227_MOESM1_ESM.pdf]

SUPPLEMENTARY INFORMATION

mTORC1 and PKB/Akt control the muscle response to denervation by regulating autophagy and HDAC4

Castets et al.

Supplementary information contains: 8 supplementary figures and 2 supplementary tables.

SUPPLEMENTARY FIGURES

Supplementary figure 1

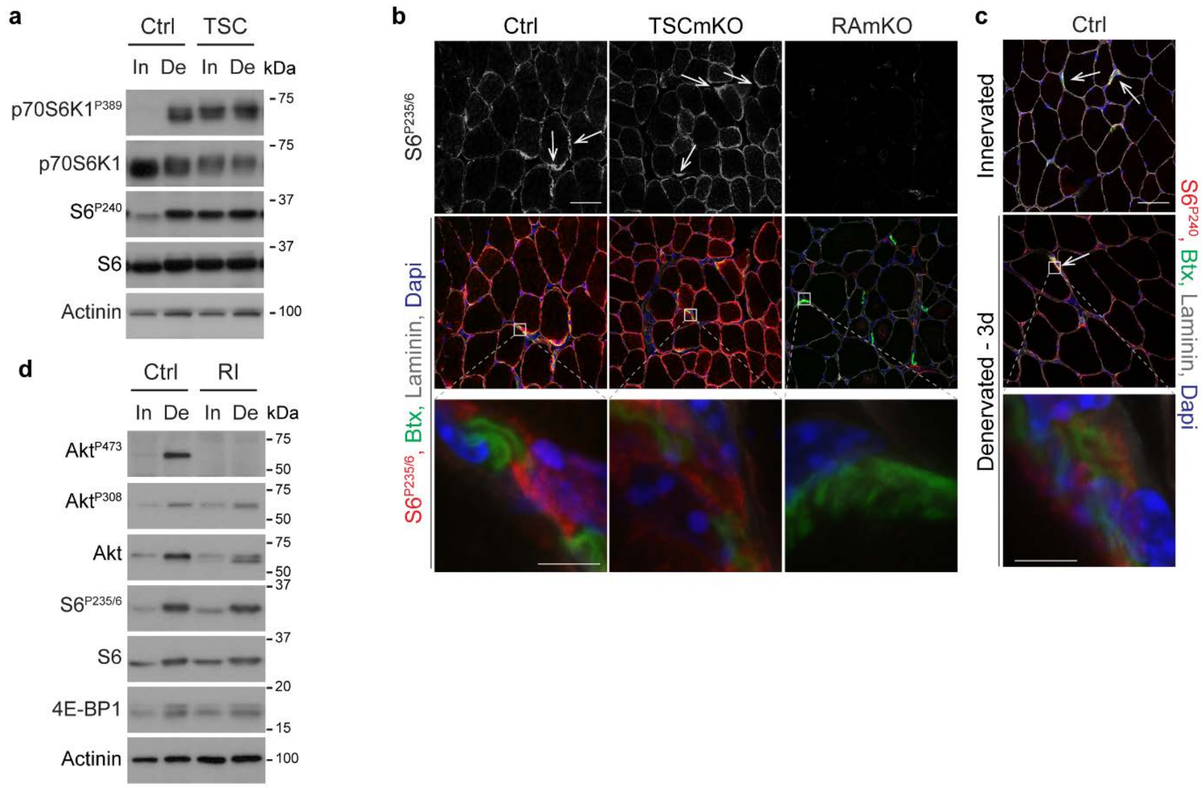

PKB/Akt and mTORC1 signaling are activated upon denervation in TA muscle.

a, Western blot analysis of mTORC1 specific targets in innervated (In) and 3-day-denervated (De) TA control (Ctrl) and TSCmKO (TSC) muscles. Actinin was used as loading control. Representative image of 4 Ctrl and 3 TSCmKO mice. **b,c** Confocal pictures of $S6^{P235/6}$ (red, **b**) or $S6^{P240}$ (red, **c**), with laminin (grey), bungarotoxin (green) and Dapi (blue) in TA denervated (3d) muscle from control, TSCmKO and RAmKO mice (**b**), and in TA innervated and denervated Ctrl muscles (**c**). Scale bar, 50 µm (5 µm for enlarged view). **d**, Western blot analysis of PKB/Akt and mTORC1 pathways in TA innervated and denervated muscles from control and RImKO (RI) mice (n=3 mice per genotype). Western blot quantifications are shown in Supplementary Table 1. Source data are provided as a Source Data File.

Supplementary Figure 2

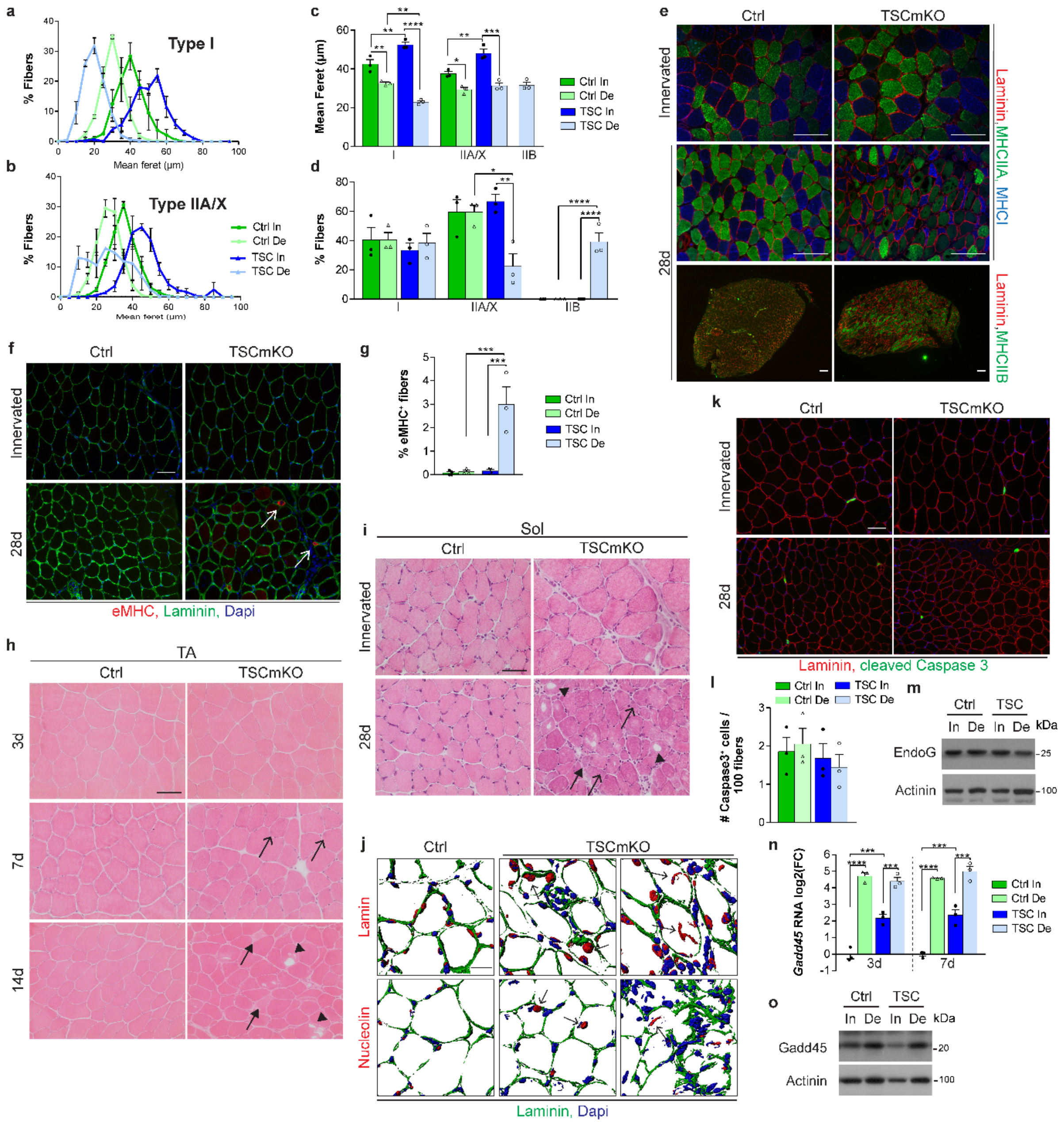

Sustained mTORC1 activation alters muscle fiber type and size upon denervation.

a-e, Size distribution for type I (**a**) and IIA/X (**b**) fibers, minimum mean fiber feret (**c**) and fiber type proportion (**d**) in control (Ctrl) and TSCmKO (TSC), innervated (In) and denervated (De, 28d) *soleus* muscles. n=3. Pictures (**e**) show MHCI (blue) and MHCIIA (green - top), or MHCIIB (green - bottom) and laminin (red) immunostaining. Scale bar, 100 µm. **f,g,** Immunostaining for embryonic MHC (eMHC, red) and laminin (green) of innervated and denervated (28d) TA muscles. Arrows point to eMHC-positive fibers. Scale bar, 50 µm. Quantification (**g**) gives the proportion of eMHC-positive fibers. n=4 (In) and

3 (De) muscles. **h,i,** HE coloration of TA muscle after 3, 7 and 14 days of denervation (**h**) and of innervated and denervated (28d) *soleus* muscles (**i**), from control and TSCmKO mice. Representative of 4 (3 and 7d) and 3 (14 and 28d) mice. Open arrows, arrows and arrowheads point to swollen nuclei, degenerating fibers and vacuoles, respectively. Scale bar, 50 µm. **j,** 3D reconstruction of lamin (red, top) and nucleolin (red, bottom) immunostaining in denervated control and TSCmKO muscles presented in Fig. 2i. Arrows indicate giant and swollen myonuclei. Scale bar, 20 µm. **k,l,** Immunostaining of denervated TA control and TSCmKO muscles (28d) for cleaved caspase 3 (green) and laminin (red). Scale bar, 50 µm. Quantification (**l**) gives the number of cleaved caspase3-positive cells for 100 fibers. n=3. **m,** Western blot analysis of Endonuclease G in innervated and 14-day-denervated TA control and TSCmKO muscles. Actinin was used as loading control. Representative of 3 mice per group. **n,o,** Transcript and protein levels of Gadd45 in innervated and denervated TA control and TSCmKO muscles. Transcript levels (n=3) are relative to *Tbp* mRNA and to Ctrl innervated muscle, and presented as the log2 fold change (FC). In **(o),** actinin was used as loading control. Representative of 4 Ctrl and 3 TSCmKO mice. All data are mean±s.e.m.; two-way ANOVA with Tukey's post-hoc test, $^{*}p<0.05$, $^{**}p<0.01$, $^{***}p<0.001$, $^{****}p<0.0001$. Western blot quantifications are shown in Supplementary Table 1. Source data are provided as a Source Data File.

Supplementary Figure 3

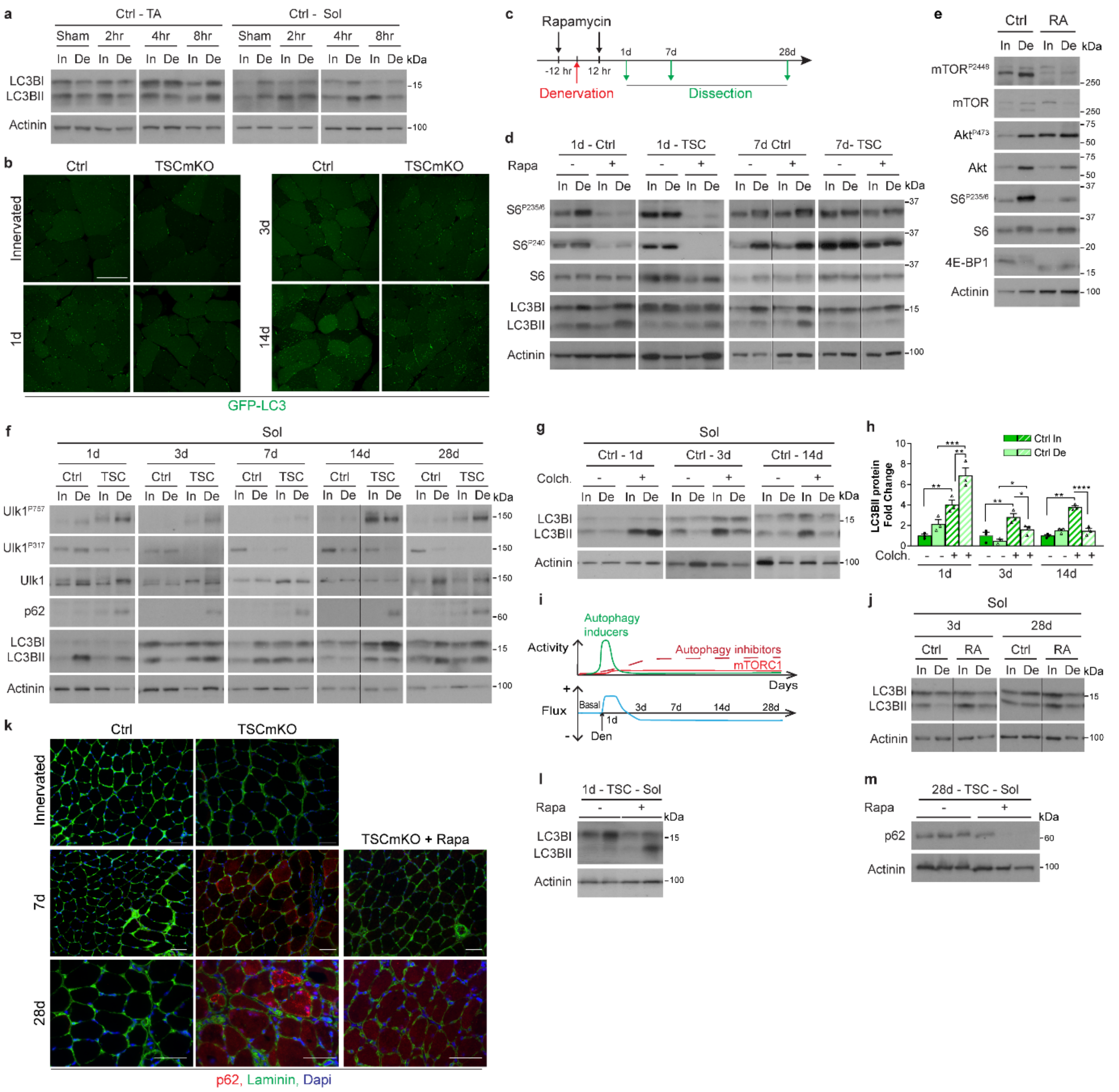

Autophagy is differentially regulated in TA and *soleus* muscles after denervation.

a, Western blot analysis of LC3B in TA and *soleus* control (Ctrl) muscles 2, 4 and 8 hr after denervation, compared to active muscle (Sham). n=3. **b,** Confocal pictures of GFP-LC3 puncta in TA innervated muscle from control and TSCmKO mice, and after 1, 3 and 14 days of denervation (representative of 4, 3 and 4 Ctrl; 2, 3 and 4 TSCmKO mice at 1, 3 and 14 days). Scale bar, 50 µm. **c,** Rapamycin treatment of control and TSCmKO mice combined with denervation**. d,** Western blot analysis of S6 and LC3B in innervated (In) and denervated (De - 1 and 7d) TA muscles from untreated (-) and rapamycin-treated

(+) control and TSCmKO (TSC) mice. n=4 (Ctrl untreated 1d) and 3 (all other conditions) mice. **e,** Western blot analysis of PKB/Akt and mTORC1 pathways in innervated and denervated TA muscles from control and RAmKO (RA) mice. n=3. **f-h,** Western blot analysis of autophagy markers in *soleus* control and TSCmKO muscles after 1 to 28 days of denervation (**f**) and upon colchicine treatment (**g**). Data in (**f**) are representative of results obtained with 4 (1d) and 3 (3-28d) Ctrl; 3 (1, 14 and 28d) and 4 (3 and 7d) TSCmKO mice. Quantification of LC3BII levels in (**g**) is given in (**h**); values are mean±s.e.m.; n=3; two-way ANOVA with Fisher's post-hoc test, *$p<0.05$, ** $p<0.01$, ***$p<0.001$, ****$p<0.0001$. **i,** Changes in autophagic flux in *soleus* muscle upon denervation. **j,** Western blot analysis of autophagy markers in innervated *soleus* control and RAmKO muscles and after 3 and 28 days of denervation. n=3 Ctrl and 4 RAmKO mice. **k,** Immunofluorescence of p62 (red) and laminin (green) in innervated *soleus* muscle and after 7 and 28 days of denervation in control and TSCmKO vehicle- and rapamycin-treated mice. Representative images from 3 Ctrl; 4/3 and 3/3 TSCmKO mice (-/+ rapamycin) at 7 and 28 days. Scale bar, 50 µm. **l,** Western blot analysis of LC3B levels in *soleus* muscle from untreated (-) and rapamycin-treated (+) TSCmKO mice, after 1 day of denervation. n=3. **m,** Western blot analysis of p62 in denervated (28d) *soleus* muscle from untreated (-) and rapamycin-treated (+) TSCmKO mice. n=3. Western blot quantifications are shown in Supplementary Table 1. Source data are provided as a Source Data File.

Supplementary Figure 4

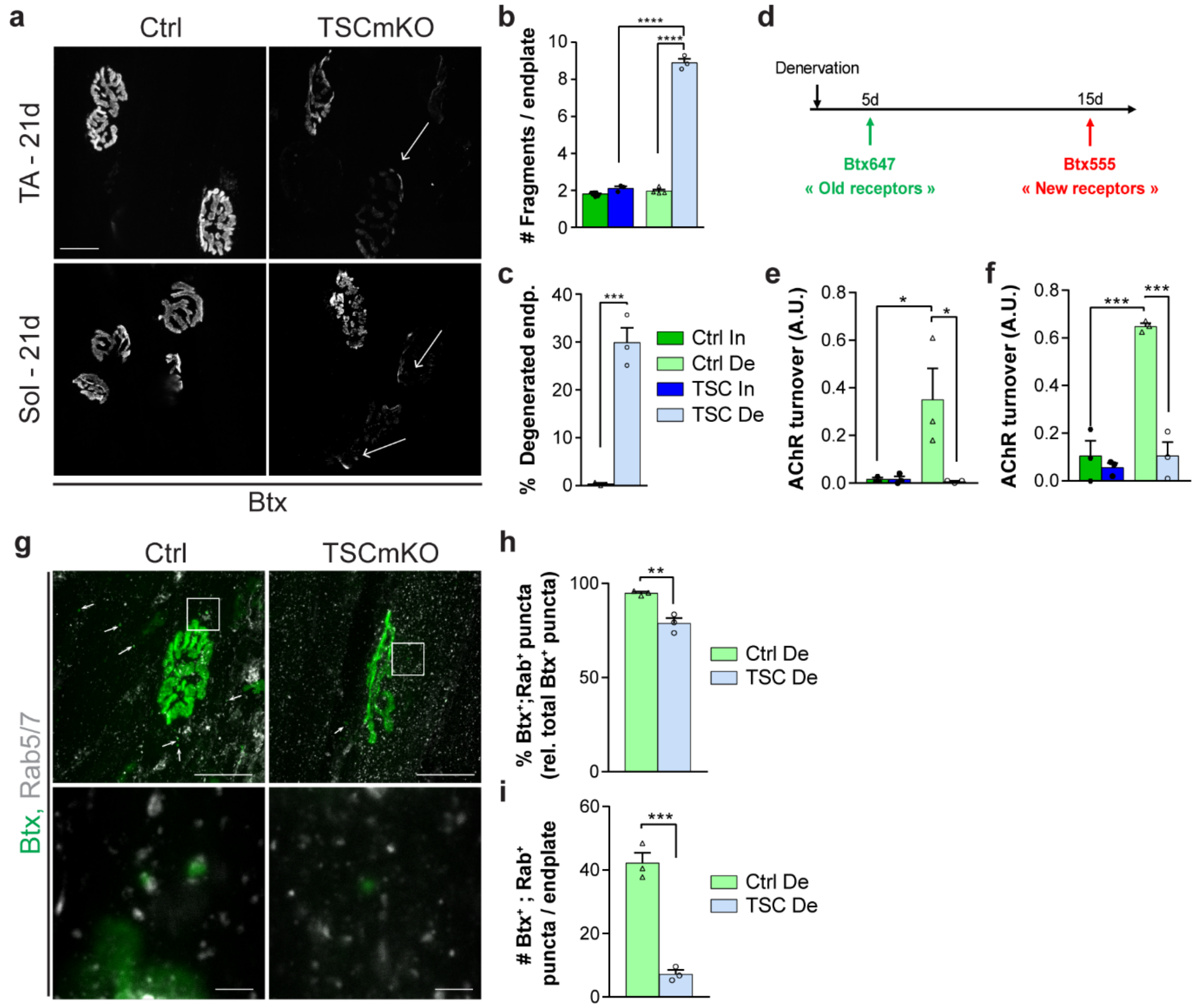

Sustained mTORC1 activation impairs endplate remodeling upon nerve injury.

a-c, Endplate degeneration and fragmentation in TA and *soleus* (Sol) muscles from TSCmKO mice after 21 days of denervation, as compared to control (Ctrl). Scale bar, 50 µm. Quantification is given for *soleus* in **b** and **c**. **b,** n=4 Ctrl and 3 TSCmKO muscles per group; **c,** n=3 mice per group. **d,** Scheme of the procedure of double labelling used to quantify AChR turnover in muscle. **e,f,** Quantification of AChR turnover in innervated (In) and denervated (De) TA muscles from control and TSCmKO (TSC) mice, using live imaging (**e**) or whole-mount muscle bundles analysis (**f**). n=3 muscles per group. **g-i,** Confocal pictures of Rab5/7 immunostaining of denervated (15d) TA control and TSCmKO muscles combined with bungarotoxin staining (old receptors). Scale bar, 20 (top) and 2 (inset) µm. Quantifications give the proportion of bungarotoxin-positive puncta co-localized with Rab5/7-positive puncta **(h)**, and the

number of endocytic vesicles containing AChR (bungarotoxin- and Rab5/7-positive puncta) per endplate **(i)**. n=3 per group. Values are mean±s.e.m.; two-way ANOVA with Tukey's post-hoc test (**b,e,f**) and two-tailed unpaired Student's *t*-test (**c,h,i**), *p<0.05, **p<0.01, ***p<0.001, ****p<0.0001. Source data are provided as a Source Data File.

Supplementary Figure 5

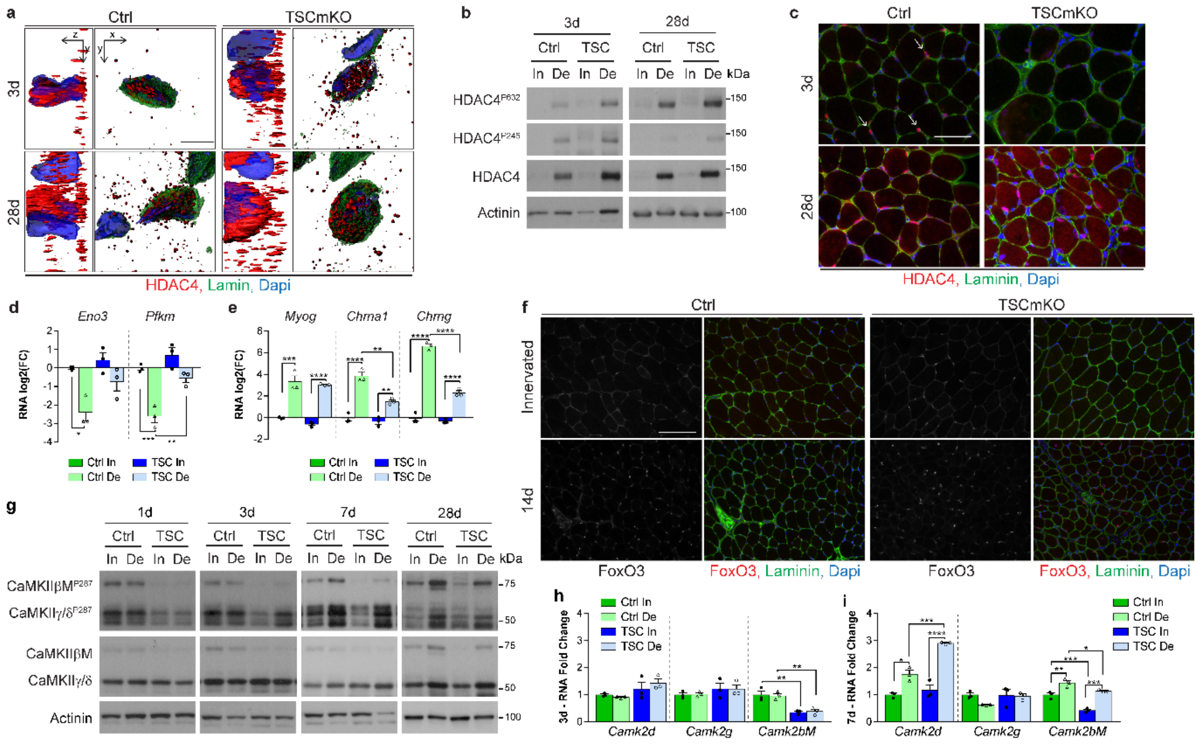

Nuclear import of HDAC4 and regulation of its target genes are impaired in TSCmKO muscle.

a, 3D reconstruction of confocal pictures of endogenous HDAC4 (red) and lamin (green) immunostaining in control (Ctrl) and TSCmKO muscles. Original pictures are shown in Fig. 5f. Scale bar, 5 µm. **b,** Western blot analysis of total and phosphorylated HDAC4 in innervated (In) *soleus* control and TSCmKO (TSC) muscles and after 3 and 14 days of denervation (De). n=3 Ctrl, 4 (3d) and 3 (28d) TSCmKO mice. **c,** Immunostaining of HDAC4 (red) and laminin (green) in denervated (3 and 28d) *soleus* control and TSCmKO muscles. Representative of 4 (3d) and 3 (28d) mice. Arrows point to HDAC4-positive myonuclei. Scale bar, 50 µm. **d,e,** mRNA levels of HDAC4 targets, *Eno* and *Pfkm* (**d**), and *Myog*, *Chrna1* and *Chrng* (**e**) in innervated and denervated *soleus* control and TSCmKO muscles. Transcript levels are relative to *Tbp* mRNA and to innervated Ctrl muscle and presented as the log2 fold change (FC). n=3. **f,** Immunostaining against FoxO3 (red) and Laminin (green) in innervated and denervated (14d) muscles from control and TSCmKO mice. Scale bar, 100 µm. **g,** Western blot analysis of total and phosphorylated CaMKII isoforms in TA innervated muscle and after 1, 3, 7 and 28 days of denervation,

in TSCmKO and control mice. n=4 (1, 3, 7d) and 3 (28d) Ctrl; 3 TSCmKO mice. **h,i,** Expression of *Camk2* transcripts in TA control and TSCmKO muscles after 3 (**h**) and 7 (**i**) days of denervation. n=3. Transcript levels were normalized to *Tbp* mRNA and to Ctrl innervated muscle. All values are mean±s.e.m.; two-way ANOVA with Tukey's post-hoc test, *p<0.05, **p<0.01, ***p<0.001, ****p<0.0001. Western blot quantifications are shown in Supplementary Table 1. Source data are provided as a Source Data File.

Supplementary Figure 6

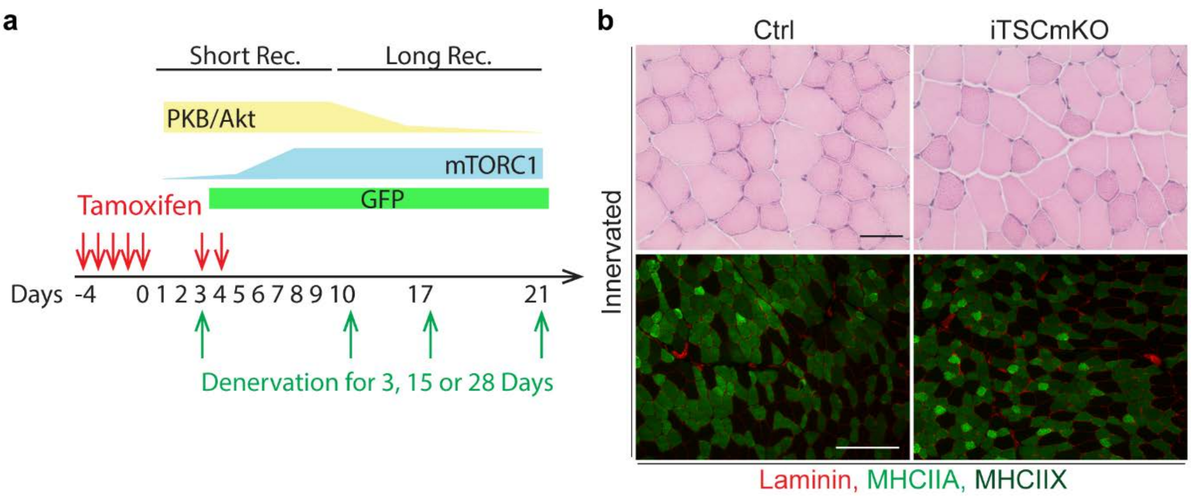

Acute mTORC1 activation does not perturb muscle homeostasis.

a, Scheme of the recombination (Rec; short *vs.* long) strategy used with iTSCmKO mice, and changes detected in the activation state of PKB/Akt and mTORC1 signaling. **b**, HE coloration (top panel) and fluorescent pictures of MHCIIA/X (bright/dark green) and laminin (red) immunostaining (bottom panel) of TA innervated muscle from control (Ctrl) and iTSCmKO (iTSC) mice (3 independent muscles per genotype). Scale bar, 50 (top) and 200 (bottom) µm.

Supplementary Figure 7

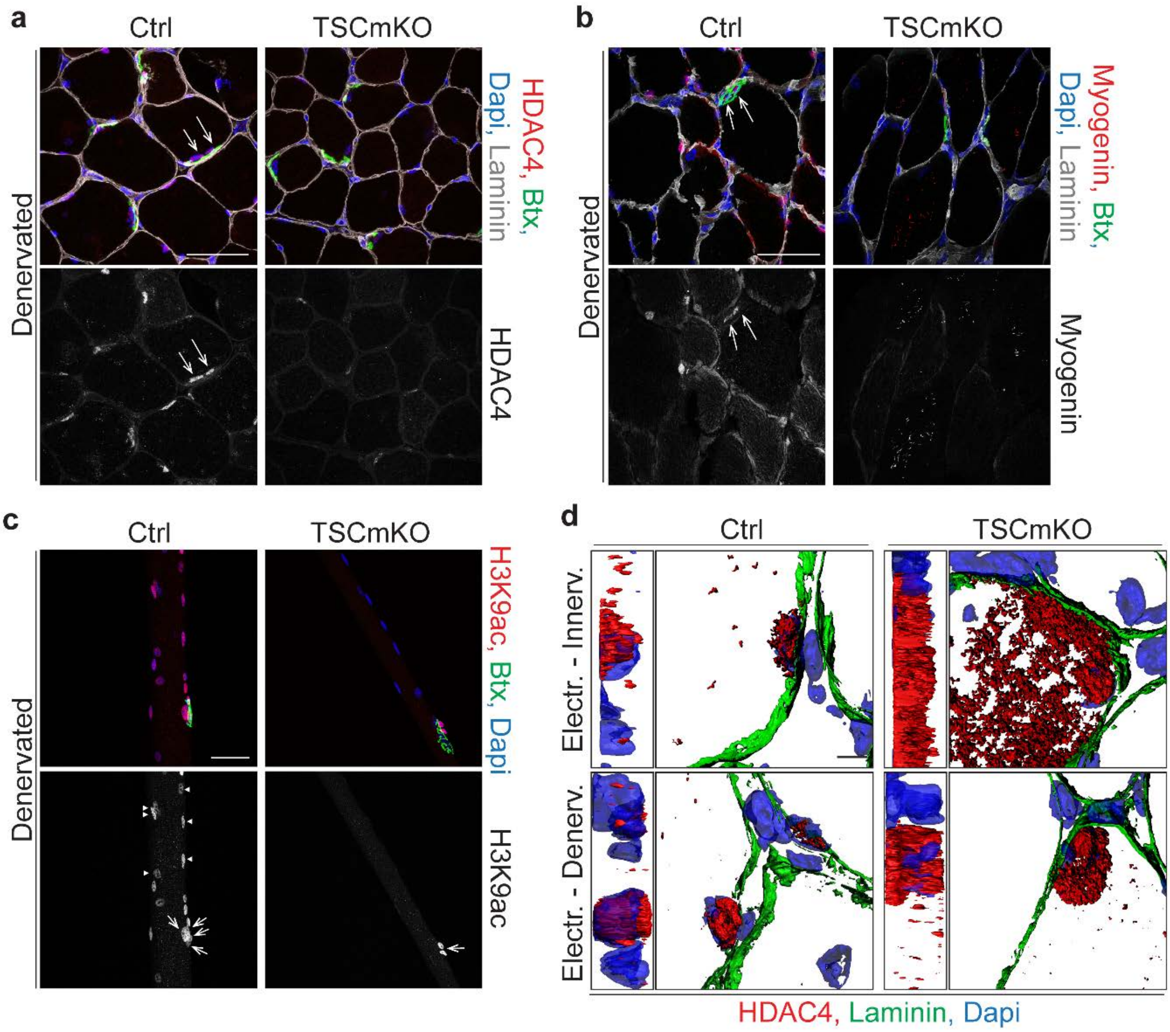

Nuclear import of HDAC4 in sub- and extra-synaptic nuclei is impaired in TSCmKO muscle.

a,b, Confocal pictures (from 3 independent assays) of HDAC4 (red, a) or myogenin (red, b), with bungarotoxin (green) and laminin (grey) on muscle sections from denervated control (Ctrl) and TSCmKO muscles. Arrows point to sub-synaptic nuclei positive for HDAC4 **(a)** or myogenin **(b).** Scale bar, 50 µm. **c,** Confocal pictures of acetylated histone H3 (Lys9, H3K9ac - red) and bungarotoxin (green) in isolated fibers from denervated control and TSCmKO muscles. Representative of 4 independent assays. Arrows and arrowheads show sub- and extra-synaptic myonuclei, respectively. Scar bar, 50 µm. **d,** 3D reconstruction of confocal pictures of electroporated HDAC4 (red) and laminin (green)

immunostaining in control and TSCmKO muscles. Original pictures are shown in Fig. 7d. Scale bar, 5 µm.

Supplementary Figure 8

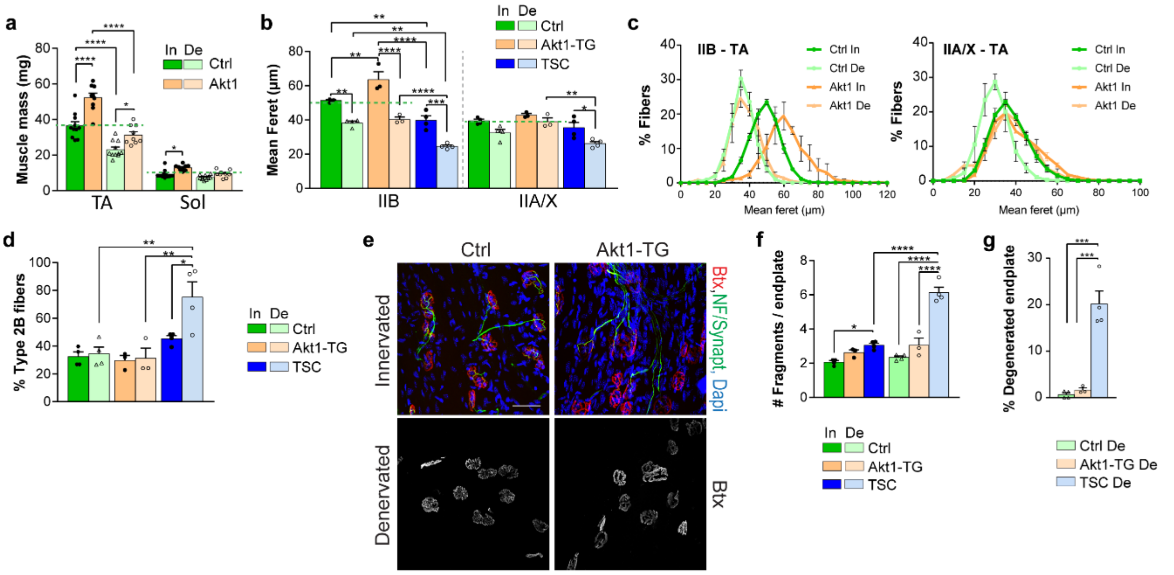

Muscle response to denervation in Akt1-TG mice.

a, Mass of innervated and denervated (14d) TA and *soleus* (Sol) muscles from control (Ctrl) and Akt1-TG mice. Mice were treated with tamoxifen one day before denervation and over the time of experiment. The dashed line indicates the initial mass of the innervated muscle. n=11 Ctrl and 9 Akt1-TG mice. **b,c,** Minimum mean fiber feret (**b**) and fiber size distribution (**c**) in innervated (In) and denervated (De, 14d) TA muscles from control, Akt1-TG and TSCmKO mice, distinguishing type IIB and IIA/X fibers. n=4 Ctrl, 3 Akt1-TG and 4 TSCmKO mice. **d,** Proportion of type IIB fibers in innervated TA muscle from control, Akt1-TG and TSCmKO mice and after 14 days denervation. n=4 Ctrl, 3 Akt1-TG and 4 TSCmKO mice. **e-g,** Fluorescent images of NMJ regions stained with bungarotoxin (Btx, red) and antibodies against neurofilament and synaptophysin (NF/Synapt, green) in innervated and denervated (14d) TA muscles from control and Akt1-TG mice (**e**). Only the bungarotoxin staining is shown for denervated muscle. Scale bar, 50 µm. Quantifications give the number of fragments per endplate (**f**) and the proportion of degenerated endplates (**g**). n=4 Ctrl, 3 Akt1-TG and 4 TSCmKO mice. All values are mean±s.e.m.; two- (**a,b,d,f**) and one- (**g**) way ANOVA with Tukey's post-hoc test, *p<0.05, **p<0.01, ***p<0.005, ****p<0.0005. Source data are provided as a Source Data File.

SUPPLEMENTARY TABLES

Supplementary Table 1

Western blot quantification

Figure 1a	Denerv. 14hr		Denerv. 1d		Denerv. 3d	
	Ctrl Inn	Ctrl Den	Ctrl Inn	Ctrl Den	Ctrl Inn	Ctrl Den
mTOR^{P2448}/mTOR	1.00±0.53	0.62±0.32	1.00±0.15	1.51±0.14*	1.00±0.03	1.90±0.61
mTOR^{P2448}/ actinin	1.00±0.64	1.00±0.62	1.00±0.03	1.54±0.10**	1.00±0.16	3.13±0.65*
mTOR pan	1.00±0.28	1.77±0.35	1.00±0.16	0.98±0.13	1.00±0.19	1.75±0.19*
S6$^{P235/6}$/S6	1.00±0.25	0.98±0.15	1.00±0.13	2.45±0.21**	1.00±0.13	4.86±0.54**
S6$^{P235/6}$/ actinin	1.00±0.33	0.87±0.20	1.00±0.18	2.06±0.25*	1.00±0.10	4.22±0.62**
S6 pan	1.00±0.21	0.92±0.23	1.00±0.06	0.85±0.07	1.00±0.04	0.91±0.21
4E-BP1 β/α	1.00±0.07	1.07±0.06	1.00±0.11	1.01±0.06	1.00±0.09	1.42±0.12*

Figure 1a	Denerv. 7d		Denerv. 14d		Denerv. 28d		Denerv. 28d	
	Ctrl Inn	Ctrl Den	Ctrl Inn	Ctrl Den	Ctrl Inn	Ctrl Den	TSCmKO Inn	TSCmKO Den
mTOR^{P2448}/mTOR	1.00±0.28	1.46±0.43	1.00±0.32	2.42±0.71	1.00±0.20	0.91±0.14	1.85±0.14$^{0.06b}$	1.72±0.49
mTOR^{P2448}/ actinin	1.00±0.22	3.95±0.53**	1.00±0.25	5.80±1.23*	1.00±0.07	2.99±0.65*	2.44±0.56$^{0.05b}$	4.69±0.25**$^{\$}$
mTOR pan	1.00±0.11	2.78±0.39*	1.00±0.08	2.47±0.32*	1.00±0.14	3.04±0.20**	1.27±0.33	2.95±0.67*
S6$^{P235/6}$/S6	1.00±0.05	1.87±0.34 $^{0.06a}$	1.00±0.18	1.99±0.23*	1.00±0.16	2.68±0.54*	2.73±0.24$^{\$}$	2.89±0.64
S6$^{P235/6}$/ actinin	1.00±0.11	4.11±0.71*	1.00±0.20	9.92±1.66**	1.00±0.25	4.57±0.31***	6.47±0.38$^{\$\$\$\$}$	6.67±0.80$^{\$}$
S6 pan	1.00±0.13	2.19±0.10**	1.00±0.24	4.62±0.25***	1.00±0.12	1.86±0.22*	2.50±0.28$^{\$\$}$	2.51±0.32
4E-BP1 β/α	1.00±0.24	1.88±0.07*	1.00±0.28	2.01±0.22*	1.00±0.10	2.04±0.50$^{0.06a}$	2.11±0.34$^{\$}$	3.63±0.30*$^{\$}$

Figure 1c	Denerv. 14hr		Denerv. 1d		Denerv. 3d	
	Ctrl Inn	Ctrl Den	Ctrl Inn	Ctrl Den	Ctrl Inn	Ctrl Den
Akt^{P473}/Akt	1.00±0.26	1.00±0.14	1.00±0.28	0.87±0.20	1.00±0.23	0.85±0.22
Akt^{P473}/ actinin	1.00±0.22	0.70±0.15	1.00±0.24	0.92±0.26	1.00±0.06	1.19±0.09
Akt^{P308}/Akt	1.00±0.23	1.19±0.05	1.00±0.36	0.64±0.07	1.00±0.40	0.71±0.20
Akt^{P308}/ actinin	1.00±0.24	0.90±0.33	1.00±0.19	0.63±0.10	1.00±0.39	1.02±0.23
Akt pan	1.00±0.26	0.72±0.28	1.00±0.08	1.01±0.13	1.00±0.27	2.34±0.21**

Figure 1c	Denerv. 7d		Denerv. 14d		Denerv. 28d		Denerv. 28d	
	Ctrl Inn	Ctrl Den	Ctrl Inn	Ctrl Den	Ctrl Inn	Ctrl Den	TSCmKO Inn	TSCmKO Den
Akt^{P473}/Akt	1.00±0.16	0.81±0.12	1.00±0.21	1.79±0.50	1.00±0.09	0.67±0.08*	0.12±0.02$^{\$\$\$\$}$	0.20±0.10$^{\$\$}$
Akt^{P473}/ actinin	1.00±0.14	2.06±0.28*	1.00±0.21	4.41±0.64**	1.00±0.04	1.88±0.02***	0.25±0.05$^{\$\$\$}$	0.52±0.19$^{\$\$\$\$}$
Akt^{P308}/Akt	1.00±0.19	1.11±0.20	1.00±0.31	1.06±0.15	1.00±0.06	0.38±0.04***	0.42±0.05$^{\$\$\$}$	0.65±0.11*$^{\$}$
Akt^{P308}/ actinin	1.00±0.18	1.44±0.19	1.00±0.44	2.31±0.24$^{0.06a}$	1.00±0.10	1.92±0.19**	0.42±0.03$^{\$}$	1.12±0.28*$^{\$}$
Akt pan	1.00±0.08	2.54±0.05****	1.00±0.19	1.99±0.07**	1.00±0.08	5.21±0.90***	1.03±0.14	1.83±0.48$^{\$\$}$

Figure 1h	Sol - Denerv. 7d		Sol - Denerv. 14d		Sol - Denerv. 28d		Sol - Denerv. 28d	
	Ctrl Inn	Ctrl Den	Ctrl Inn	Ctrl Den	Ctrl Inn	Ctrl Den	TSCmKO Inn	TSCmKO Den
Akt^{P473}/Akt	1.00±0.19	0.71±0.17	1.00±0.15	1.11±0.21	1.00±0.35	1.15±0.24	0.37±0.06	0.68±0.28
Akt^{P473}/ actinin	1.00±0.12	0.78±0.03	1.00±0.07	1.49±0.19	1.00±0.17	1.47±0.27	0.16±0.01$^{\$\$}$	0.71±0.15$^{\$}$
Akt pan	1.00±0.26	1.10±0.20	1.00±0.13	1.42±0.33	1.00±0.24	1.20±0.24	0.39±0.07	1.23±0.41
S6$^{P235/6}$/S6	1.00±0.30	1.67±0.37	1.00±0.16	0.85±0.13	1.00±0.30	0.91±0.18	2.02±0.42$^{0.06b}$	2.01±0.40$^{\$}$
S6$^{P235/6}$/ actinin	1.00±0.21	1.21±0.22	1.00±0.11	1.42±0.29	1.00±0.33	1.29±0.24	2.23±0.62	3.60±0.36*$^{\$\$}$
S6 pan	1.00±0.29	0.65±0.15	1.00±0.16	1.57±0.19	1.00±0.06	1.45±0.18	1.10±0.12	1.91±0.30*
4E-BP1 β/α	1.00±0.14	0.94±0.04	1.00±0.07	1.06±0.08	1.00±0.15	0.94±0.14	5.37±0.53$^{\$\$\$\$}$	4.26±0.35*$^{\$\$\$}$

Figure S1a	Denerv. 3d		Denerv. 3d	
	Ctrl Inn	Ctrl Den	TSCmKO Inn	TSCmKO Den
p70S6K1^{P389}/p70S6K1	1.00±0.12	32.37±8.06*	36.04±8.88$^{\$}$	35.83±14.38
p70S6K1^{P389}/ actinin	1.00±0.15	27.12±8.37**	26.38±2.28$^{\$\$}$	27.30±3.25
p70S6K1 / actinin	1.00±0.12	0.90±0.20	0.81±0.17	0.94±0.24
S6^{P240}/S6	1.00±0.30	4.02±0.72**	3.83±0.56$^{\$\$}$	3.63±0.80
S6^{P240}/ actinin	1.00±0.29	4.14±0.60**	5.40±1.24$^{\$\$}$	4.71±0.51
S6 pan	1.00±0.03	1.04±0.10	1.38±0.17	1.42±0.38

Figure S1d	Denerv. 14d		Denerv. 14d	
	Ctrl Inn	Ctrl Den	RImKO Inn	RImKO Den
Akt^{P473}/Akt	1.00±0.18	1.16±0.18	0.31±0.08$^{\$\$}$	0.20±0.03$^{\$\$\$}$
Akt^{P473}/ actinin	1.00±0.19	5.70±0.56****	0.28±0.06	0.52±0.07$^{\$\$\$\$}$
Akt^{P308}/Akt	1.00±0.37	0.25±0.06	1.01±0.29	0.72±0.23
Akt^{P308}/ actinin	1.00±0.11	1.42±0.26	1.14±0.32	2.12±0.48
Akt pan	1.00±0.33	4.54±0.33****	0.97±0.37	2.44±0.35*$^{\$\$}$
S6$^{P235/6}$/S6	1.00±0.12	3.25±0.41*	1.16±0.02	4.57±1.22**
S6$^{P235/6}$/ actinin	1.00±0.29	7.92±1.39**	1.38±0.27	7.60±1.91**
S6 pan	1.00±0.39	2.21±0.14*	1.10±0.23	1.58±0.22
4E-BP1 β/α	1.00±0.06	2.15±0.38*	0.86±0.24	1.47±0.25

Figure S2m	Denerv. 14d		Denerv. 14d	
	Ctrl Inn	Ctrl Den	TSCmKO Inn	TSCmKO Den
EndoG / actinin	1.00±0.28	1.23±0.14	1.41±0.36	0.96±0.09

Figure S2o	Denerv. 3d		Denerv. 3d	
	Ctrl Inn	Ctrl Den	TSCmKO Inn	TSCmKO Den
Gadd45 / actinin	1.00±0.13	1.83±0.18	1.50±0.17	2.35±0.69

Figure 3a	Denerv. 14hr		Denerv. 14hr		Denerv. 1d		Denerv. 1d	
	Ctrl Inn	Ctrl Den	TSCmKO Inn	TSCmKO Den	Ctrl Inn	Ctrl Den	TSCmKO Inn	TSCmKO Den
$Ulk1^{P757}/Ulk1$	1.00±0.15	1.34±0.26	3.40±0.44[$$$]	4.07±0.60[$$$]	1.00±0.15	1.29±0.35	8.21±0.83[$$$$]	6.84±1.42[$$$]
$Ulk1^{P757}$/ actinin	1.00±0.40	0.92±0.28	9.42±1.72[$$$$]	8.50±1.28[$$$]	1.00±0.06	1.18±0.18	4.64±0.28[$$$$]	4.13±0.81[$$$]
$Ulk1^{P317}/Ulk1$	1.00±0.23	1.04±0.35	0.14±0.03[$]	0.23±0.03[$]	1.00±0.16	1.21±0.16	0.88±0.20	0.95±0.21
$Ulk1^{P317}$/actinin	1.00±0.13	0.71±0.07*	0.46±0.02[$$]	0.61±0.05	1.00±0.18	1.12±0.13	0.49±0.12[$]	0.57±0.15[$]
p62	1.00±0.40	1.50±0.63	9.10±1.38[$$]	10.42±3.95[$$]	1.00±0.28	1.05±0.34	7.71±0.74[$$$$]	8.43±0.73[$$$$]
Beclin1	1.00±0.39	0.88±0.34	1.76±0.36	1.45±0.23	1.00±0.09	1.11±0.21	2.31±0.38[$$]	2.37±0.08[$$]
LC3BII/LC3BI	1.00±0.22	1.14±0.18	0.40±0.08[$]	0.23±0.00[$$]	1.00±0.08	1.10±0.07	0.64±0.05[$]	0.45±0.11[$$$]
LC3BII/actinin	1.00±0.39	0.74±0.21	0.50±0.06	0.33±0.05	1.00±0.17	0.99±0.12	0.86±0.21	0.52±0.16[0.07b]

Figure 3a	Denerv. 3d		Denerv. 3d		Denerv. 7d		Denerv. 7d	
	Ctrl Inn	Ctrl Den	TSCmKO Inn	TSCmKO Den	Ctrl Inn	Ctrl Den	TSCmKO Inn	TSCmKO Den
$Ulk1^{P757}/Ulk1$	1.00±0.24	4.19±0.85*	4.04±0.17[$]	5.34±1.39	1.00±0.21	2.22±0.50	5.79±2.06[$]	5.02±2.27
$Ulk1^{P757}$/ actinin	1.00±0.07	6.90±0.93**	7.00±1.51[$$]	9.59±1.83	1.00±0.18	3.06±0.51*	6.74±0.92[$$$$]	9.72±0.36**[$$$$]
$Ulk1^{P317}/Ulk1$	1.00±0.15	0.31±0.05**	0.48±0.10[$]	0.42±0.22	1.00±0.22	0.44±0.08	1.12±0.47	0.54±0.35
$Ulk1^{P317}$/actinin	1.00±0.09	0.53±0.11	0.85±0.31	0.75±0.35	1.00±0.16	0.63±0.08	1.28±0.20	0.91±0.28
p62	1.00±0.14	8.29±1.41	5.28±0.93	28.11±7.36***[$$]	1.00±0.07	6.30±1.57	13.36±4.24	70.19±28.46*[$$]
Beclin1	1.00±0.24	1.22±0.20	2.17±0.68[$]	2.12±0.14	1.00±0.24	2.05±0.33[0.08a]	3.12±0.36[$$]	4.29±0.75[$$]
LC3BII/LC3BI	1.00±0.30	0.88±0.13	0.87±0.09	0.85±0.32	1.00±0.22	0.89±0.08	0.42±0.04[$]	0.51±0.03[0.08b]
LC3BII/actinin	1.00±0.25	1.11±0.15	1.09±0.21	1.11±0.26	1.00±0.20	1.60±0.22*	0.44±0.01[0.05b]	1.10±0.09*[0.07b]

Figure 3a	Denerv. 14d		Denerv. 14d		Denerv. 28d		Denerv. 28d	
	Ctrl Inn	Ctrl Den	TSCmKO Inn	TSCmKO Den	Ctrl Inn	Ctrl Den	TSCmKO Inn	TSCmKO Den
$Ulk1^{P757}/Ulk1$	1.00±0.25	2.98±0.89	8.69±1.90[$]	11.75±2.95[$$]	1.00±0.34	0.85±0.11	3.92±0.73	5.85±3.24[0.06b]
$Ulk1^{P757}$/ actinin	1.00±0.30	4.04±0.96	5.11±0.58[0.05b]	9.08±2.39[$]	1.00±0.25	2.09±0.23	14.14±5.56[$]	19.19±4.92[$]
$Ulk1^{P317}/Ulk1$	1.00±0.20	0.38±0.13*	0.59±0.12[0.05b]	0.04±0.01*	1.00±0.34	0.30±0.10*	0.44±0.06	0.08±0.01
$Ulk1^{P317}$/actinin	1.00±0.32	0.46±0.07[0.05b]	0.35±0.12[$]	0.04±0.01	1.00±0.24	0.68±0.14	1.39±0.30	0.37±0.13*
p62	1.00±0.09	1.88±0.44	15.41±2.14[0.08b]	37.67±10.26*[$$]	1.00±0.29	2.70±0.73	13.70±6.21[$]	17.27±4.26*
Beclin1	1.00±0.27	2.48±0.29*	1.79±0.22	2.70±0.76	1.00±0.06	2.44±0.46	4.17±1.28[$]	7.06±0.84*[$$]
LC3BII/LC3BI	1.00±0.11	1.58±0.15**	0.56±0.06[$]	0.73±0.02[$$$]	1.00±0.17	1.46±0.09*	0.52±0.14[$]	0.84±0.11[$$]
LC3BII/actinin	1.00±0.12	5.54±1.56**	0.51±0.06	1.33±0.36[$$]	1.00±0.15	3.43±0.45***	0.70±0.12	1.41±0.42[$$]

Figure 3b	Denerv. 1d		Denerv. 1d + Colchicine		Denerv. 3d		Denerv. 3d + Colchicine	
	Ctrl Inn	Ctrl Den	Ctrl Inn	Ctrl Den	Ctrl Inn	Ctrl Den	Ctrl Inn	Ctrl Den
LC3BII/LC3BI	1.00±0.18	1.25±0.23	3.64±0.41[$$$]	3.08±0.36[$$]	1.00±0.26	0.89±0.07	3.02±0.39[$$]	1.85±0.32*[$]
LC3BII/actinin	1.00±0.30	1.10±0.11	2.41±0.26[$]	2.02±0.45[0.06b]	1.00±0.01	0.85±0.06	3.62±0.66[$$$]	2.98±0.30[$$]

Figure 3b	Denerv. 14d		Denerv. 14d + Colchicine		Denerv. 14d		Denerv. 14d + Colchicine	
	Ctrl Inn	Ctrl Den	Ctrl Inn	Ctrl Den	TSCmKO Inn	TSCmKO Den	TSCmKO Inn	TSCmKO Den
LC3BII/LC3BI	1.00±0.10	1.45±0.23	6.65±0.98[$$$$]	6.15±0.43[$$$]	1.00±0.14	1.86±0.18*	1.87±0.35[$]	1.82±0.07
LC3BII/actinin	1.00±0.11	2.92±0.57*	4.86±0.73[$$$]	6.72±0.54*[$$]	1.00±0.09	2.01±0.26*	1.97±0.30[$]	2.86±0.22*[$]

Figure 3g	Denerv. 3d		Denerv. 3d		Denerv. 28d		Denerv. 28d	
	Ctrl Inn	Ctrl Den	RAmKO Inn	RAmKO Den	Ctrl Inn	Ctrl Den	RAmKO Inn	RAmKO Den
p62	1.00±0.18	9.32±1.96***	1.36±0.39	3.22±0.94[$$]	1.00±0.27	2.57±0.58[0.05a]	0.92±0.16	2.15±0.71
LC3BII/LC3BI	1.00±0.10	1.24±0.13	2.59±0.37[$]	4.34±0.55**[$$$]	1.00±0.17	2.23±0.47*	3.04±0.09[$$$]	3.83±0.10[0.06a$$]
LC3BII /actinin	1.00±0.09	1.07±0.14	3.35±0.81[$]	4.85±0.70[$$]	1.00±0.27	4.01±0.63	2.92±0.71	5.25±2.01

Figure 3k	Denerv. 28d - TSC TA	
	-	+ rapamycin
p62	1.00±0.27	1.69±0.34

Figure S3a	Sham - TA		Denerv. 2 hr - TA		Denerv. 4 hr - TA		Denerv. 8 hr - TA	
	Ctrl left	Ctrl right	Ctrl Inn	Ctrl Den	Ctrl Inn	Ctrl Den	Ctrl Inn	Ctrl Den
LC3BII/LC3BI	1.00±0.11	0.84±0.09	1.00±0.15	1.08±0.05	1.00±0.17	0.84±0.13	1.00±0.05	1.15±0.04
LC3BII	1.00±0.11	0.79±0.05	1.00±0.14	1.07±0.11	1.00±0.04	1.04±0.27	1.00±0.08	1.12±0.14

Figure S3a	Sham - sol		Denerv. 2 hr - sol		Denerv. 4 hr - sol		Denerv. 8 hr - sol	
	Ctrl left	Ctrl right	Ctrl Inn	Ctrl Den	Ctrl Inn	Ctrl Den	Ctrl Inn	Ctrl Den
LC3BII/LC3BI	1.00±0.25	0.93±0.33	1.00±0.21	1.36±0.63	1.00±0.12	1.41±0.26	1.00±0.13	0.92±0.22
LC3BII	1.00±0.17	1.42±0.06	1.00±0.12	0.98±0.30	1.00±0.43	1.21±0.05	1.00±0.08	1.02±0.23

Figure S3d	Denerv. 1d		Denerv. 1d + rapamycin		Denerv. 1d		Denerv. 1d + rapamycin	
	Ctrl Inn	Ctrl Den	Ctrl Inn	Ctrl Den	TSCmKO Inn	TSCmKO Den	TSCmKO Inn	TSCmKO Den
$S6^{P235/6}/S6$	1.00±0.23	2.60±0.44**	0.70±0.08	0.86±0.40[$$]	1.00±0.24	0.91±0.22	0.19±0.10[$$]	0.08±0.02[$$]
$S6^{P235/6}$/actinin	1.00±0.26	2.17±0.18**	0.59±0.23	0.39±0.19[$$$]	1.00±0.04	1.06±0.11	0.10±0.02[$$$$]	0.07±0.01[$$$$]
$S6^{P240}/S6$	1.00±0.24	2.07±0.29**	0.20±0.06[$]	0.37±0.06[$$$]	1.00±0.32	0.75±0.25	0.04±0.02[$]	0.03±0.02[$]
$S6^{P240}$/ actinin	1.00±0.19	1.76±0.10**	0.14±0.03[$$$]	0.17±0.03[$$$$]	1.00±0.15	0.90±0.20	0.02±0.00[$$$]	0.03±0.01[$$]
LC3BII / LC3I	1.00±0.11	1.16±0.08	1.23±0.10	1.49±0.07[$]	1.00±0.12	1.02±0.18	2.10±0.12[$$$]	2.79±0.12**[$$$$]
LC3BII / actinin	1.00±0.47	1.08±0.29	1.26±0.30	1.50±0.61	1.00±0.22	1.03±0.25	1.58±0.71	1.96±0.50

1

Figure S3d	Denerv. 7d		Denerv. 7d + rapamycin		Denerv. 7d		Denerv. 7d + rapamycin	
	Ctrl Inn	Ctrl Den	Ctrl Inn	Ctrl Den	TSCmKO Inn	TSCmKO Den	TSCmKO Inn	TSCmKO Den
S6^{P235}/S6	1.00±0.14	1.62±0.15**	1.39±0.13	1.64±0.06	1.00±0.32	1.09±0.09	1.67±0.41	1.00±0.11
S6$^{P235/6}$/actinin	1.00±0.26	1.79±0.21	1.26±0.44	1.59±0.19	1.00±0.29	1.00±0.16	0.93±0.08	2.60±0.99
S6^{P240}/S6	1.00±0.33	2.46±0.76	3.12±0.83$^{\$}$	4.14±0.43	1.00±0.16	0.99±0.23	1.32±0.23	0.57±0.18*
S6^{P240}/ actinin	1.00±0.06	3.04±0.70**	2.86±0.19$^{\$\$}$	4.75±0.05**$^{\$}$	1.00±0.14	0.83±0.06	0.75±0.07	1.27±0.52
LC3BII / LC3I	1.00±0.37	0.66±0.16	0.44±0.12	0.88±0.17	1.00±0.12	0.69±0.04	0.89±0.11	0.68±0.13
LC3BII / actinin	1.00±0.12	2.09±0.19$^{0.06a}$	1.35±0.44	4.36±0.53***$^{\$\$}$	1.00±0.09	0.81±0.11	1.04±0.10	1.24±0.29

Figure S3e	Denerv. 14d		Denerv. 14d	
	Ctrl Inn	Ctrl Den	RAmKO Inn	RAmKO Den
mTOR^{P2448}/mTOR	1.00±0.11	1.85±0.25**	0.17±0.07$^{\$\$}$	0.30±0.07$^{\$\$\$\$}$
mTOR^{P2448}/actinin	1.00±0.25	3.68±0.66***	0.31±0.08	0.20±0.04$^{\$\$\$}$
mTOR pan	1.00±0.16	2.06±0.25*	2.31±0.51$^{\$}$	0.73±0.18**$^{\$}$
Akt^{P473}/Akt	1.00±0.27	0.62±0.09	3.93±0.53$^{\$\$\$}$	1.33±0.07***
Akt^{P473}/actinin	1.00±0.27	3.36±0.68*	3.39±0.23$^{\$}$	4.71±0.79
Akt pan	1.00±0.11	5.20±0.47***	0.89±0.19	3.52±0.71**$^{\$}$
S6^{P235}/S6	1.00±0.20	2.63±0.63*	0.43±0.08	0.63±0.17$^{\$\$}$
S6^{P235}/actinin	1.00±0.35	4.19±0.28****	0.22±0.02$^{\$}$	0.52±0.11$^{\$\$\$\$}$
S6 pan	1.00±0.10	2.19±0.17**	0.96±0.17	1.55±0.30
4E-BP1 β/α	1.00±0.11	1.69±0.23**	0.29±0.05$^{\$\$}$	0.26±0.11$^{\$\$\$\$}$

Figure S3f	Sol - Denerv. 1d		Sol - Denerv. 1d		Sol - Denerv. 3d		Sol - Denerv. 3d	
	Ctrl Inn	Ctrl Den	TSCmKO Inn	TSCmKO Den	Ctrl Inn	Ctrl Den	TSCmKO Inn	TSCmKO Den
Ulk1^{P757}/Ulk1	1.00±0.55	1.55±0.48	12.37±3.03$^{\$\$}$	12.85±3.18$^{\$\$}$	1.00±0.22	1.53±0.48	3.08±0.67$^{\$}$	2.34±0.70
Ulk1^{P757}/actinin	1.00±0.48	0.92±0.21	10.29±3.88$^{\$\$}$	9.99±1.68$^{\$\$}$	1.00±0.06	1.28±0.17	8.58±1.53$^{\$\$}$	5.24±2.27
Ulk1^{P317}/Ulk1	1.00±0.26	1.02±0.29	0.59±0.21	0.53±0.16	1.00±0.38	0.19±0.10**	0.12±0.07$^{\$\$}$	0.02±0.01
Ulk1^{P317}/actinin	1.00±0.18	0.63±0.16	0.35±0.03$^{\$}$	0.44±0.16	1.00±0.27	0.18±0.06**	0.37±0.19$^{\$}$	0.04±0.02
p62	1.00±0.14	1.00±0.20	2.99±0.78$^{\$\$}$	5.08±0.35**$^{\$\$\$\$}$	1.00±0.18	0.97±0.04	1.50±0.08	6.03±1.91*$^{\$}$
LC3BII/LC3BI	1.00±0.13	2.88±0.36**	1.86±0.40	1.79±0.53$^{0.05b}$	1.00±0.12	0.48±0.11	1.06±0.12	1.23±0.23 $^{\$}$
LC3BII	1.00±0.15	2.09±0.30*	1.83±0.22	1.83±0.64	1.00±0.38	0.78±0.16	1.79±0.58	2.09±1.14

Figure S3f	Sol - Denerv. 7d		Sol - Denerv. 7d		Sol - Denerv. 14d		Sol - Denerv. 14d	
	Ctrl Inn	Ctrl Den	TSCmKO Inn	TSCmKO Den	Ctrl Inn	Ctrl Den	TSCmKO Inn	TSCmKO Den
Ulk1^{P757}/Ulk1	1.00±0.23	0.93±0.24	2.48±0.43$^{\$\$}$	2.25±0.11$^{\$\$}$	1.00±0.07	1.35±0.37	8.35±2.04$^{\$\$}$	8.31±1.87$^{\$\$}$
Ulk1^{P757}/actinin	1.00±0.16	1.42±0.70	3.07±0.99$^{0.05b}$	4.42±0.32$^{\$}$	1.00±0.09	0.98±0.11	4.54±0.91$^{\$\$}$	5.24±1.01$^{\$\$}$
Ulk1^{P317}/Ulk1	1.00±0.34	0.05±0.00**	0.08±0.02$^{\$\$\$}$	0.01±0.00	1.00±0.28	0.32±0.10*	0.71±0.10	0.22±0.08$^{0.06a}$
Ulk1^{P317}/actinin	1.00±0.22	0.06±0.02****	0.10±0.03$^{\$\$\$\$}$	0.01±0.01	1.00±0.38	0.25±0.09*	0.38±0.07$^{0.05b}$	0.15±0.09
p62	1.00±0.29	0.82±0.04	3.00±0.55	14.31±4.23**$^{\$\$}$	1.00±0.31	1.23±1.04	4.03±1.32	32.10±8.33**$^{\$\$\$}$
LC3BII/LC3BI	1.00±0.19	1.01±0.12	1.01±0.07	0.64±0.08*$^{\$}$	1.00±0.05	0.70±0.21	1.07±0.02	0.76±0.03
LC3BII	1.00±0.25	1.72±0.39	4.50±1.02$^{\$}$	4.64±1.23	1.00±0.06	1.04±0.08	2.20±0.23$^{0.05b}$	3.73±0.95$^{\$\$}$

Figure S3f	Sol - Denerv. 28d		Sol - Denerv. 28d	
	Ctrl Inn	Ctrl Den	TSCmKO Inn	TSCmKO Den
Ulk1^{P757}/Ulk1	1.00±0.26	1.81±0.48	12.48±4.01$^{\$}$	5.77±3.57
Ulk1^{P757}/actinin	1.00±0.24	1.84±0.29	7.59±2.05$^{\$\$}$	9.56±1.63$^{\$\$}$
Ulk1^{P317}/Ulk1	1.00±0.18	0.38±0.13**	0.32±0.14$^{\$\$}$	0.10±0.04
Ulk1^{P317}/actinin	1.00±0.25	0.36±0.11*	0.14±0.03$^{\$\$}$	0.20±0.05
p62	1.00±0.14	1.21±0.57	2.80±0.57	34.24±17.60*$^{\$}$
LC3BII/LC3BI	1.00±0.11	0.96±0.20	0.71±0.12	0.55±0.10$^{0.06b}$
LC3BII	1.00±0.39	1.25±0.43	0.73±0.35	0.98±0.11

Figure S3g	Sol - Denerv. 1d		Sol - Denerv. 1d + Colchicine		Sol - Denerv. 3d		Sol - Denerv. 3d + Colchicine	
	Ctrl Inn	Ctrl Den	Ctrl Inn	Ctrl Den	Ctrl Inn	Ctrl Den	Ctrl Inn	Ctrl Den
LC3BII/LC3BI	1.00±0.21	1.90±0.18	2.47±0.36	8.10±3.41*$^{\$}$	1.00±0.13	0.53±0.03*	1.60±0.21$^{\$}$	0.78±0.08**
LC3BII/actinin	1.00±0.15	2.14±0.42	4.02±0.44$^{\$\$}$	6.88±0.73**$^{\$\$\$}$	1.00±0.34	0.50±0.15	2.80±0.37$^{\$\$}$	1.62±0.29*$^{\$}$

Figure S3g	Sol - Denerv. 14d		Sol - Denerv. 14d + Colchicine	
	Ctrl Inn	Ctrl Den	Ctrl Inn	Ctrl Den
LC3BII/LC3BI	1.00±0.23	0.79±0.12	2.24±0.12$^{\$\$\$}$	0.97±0.13***
LC3BII/actinin	1.00±0.10	1.55±0.15$^{0.05a}$	3.74±0.16$^{\$\$\$\$}$	1.46±0.24****

Figure S3j	Sol - Denerv. 3d		Sol - Denerv. 3d		Sol - Denerv. 28d		Sol - Denerv. 28d	
	Ctrl Inn	Ctrl Den	RAmKO Inn	RAmKO Den	Ctrl Inn	Ctrl Den	RAmKO Inn	RAmKO Den
LC3BII/LC3BI	1.00±0.16	0.63±0.14$^{0.07a}$	1.20±0.12	0.73±0.08*	1.00±0.09	0.69±0.04**	1.03±0.04	0.82±0.05*
LC3BII/actinin	1.00±0.19	0.64±0.30	1.00±0.09	0.78±0.24	1.00±0.22	0.30±0.09**	0.67±0.01$^{\$}$	0.44±0.03

Figure S3l	Denerv. 1d		Denerv. 1d + rapamycin	
	TSCmKO Inn	TSCmKO Den	TSCmKO Inn	TSCmKO Den
LC3BII/LC3BI	1.00±0.24	1.20±0.03	3.38±0.81$^{\$\$}$	5.64±0.31**$^{\$\$\$}$
LC3BII/actinin	1.00±0.24	1.66±0.30	1.46±0.14	3.15±0.24**$^{\$\$}$

Figure S3m	Denerv. 28d - TSC soleus	
	-	+ rapamycin
p62	1.00±0.09	0.30±0.18$^{\$}$

Figure5a	Denerv. 1d		Denerv. 1d		Denerv. 3d		Denerv. 3d	
	Ctrl Inn	Ctrl Den	TSCmKO Inn	TSCmKO Den	Ctrl Inn	Ctrl Den	TSCmKO Inn	TSCmKO Den
HDAC4^{P632}/HDAC4	1.00±0.49	1.04±0.28	0.66±0.37	1.66±0.29	1.00±0.08	1.35±0.30	1.63±0.39	1.36±0.33
HDAC4^{P632}/Actinin	1.00±0.41	1.52±0.49	0.79±0.48	1.75±0.10	1.00±0.22	5.62±0.46****	1.47±0.24	4.36±0.44***$
HDAC4^{P246}/HDAC4	1.00±0.21	0.83±0.15	0.77±0.21	1.11±0.28	1.00±0.21	0.41±0.10*	1.77±0.10$$	0.86±0.16**
HDAC4^{P246}/Actinin	1.00±0.18	1.06±0.21	0.80±0.23	1.05±0.26	1.00±0.16	4.80±1.31**	2.71±0.50	6.36±0.51*
HDAC4 pan	1.00±0.14	1.20±0.10	0.95±0.06	0.95±0.17	1.00±0.18	10.91±1.20****	1.41±0.19	7.66±1.86**

Figure5a	Denerv. 7d		Denerv. 7d		Denerv. 14d		Denerv. 14d	
	Ctrl Inn	Ctrl Den	TSCmKO Inn	TSCmKO Den	Ctrl Inn	Ctrl Den	TSCmKO Inn	TSCmKO Den
HDAC4^{P632}/HDAC4	1.00±0.21	1.02±0.24	1.47±0.49	2.03±0.78	1.00±0.45	0.79±0.25	0.47±0.29	0.65±0.21
HDAC4^{P632}/Actinin	1.00±0.18	7.58±1.13**	1.15±0.42	8.45±3.10**	1.00±0.27	8.70±2.09**	0.83±0.37	6.50±1.76*
HDAC4^{P246}/HDAC4	1.00±0.11	0.43±0.08**	0.99±0.15	0.52±0.14*	1.00±0.18	1.29±0.19	0.74±0.08	1.10±0.16
HDAC4^{P246}/Actinin	1.00±0.19	5.53±1.24$^{0.05a}$	0.81±0.02	7.00±3.34*	1.00±0.13	15.17±3.62*	1.43±0.12	12.66±5.67*
HDAC4 pan	1.00±0.13	12.77±1.22****	0.88±0.16	12.42±2.69***	1.00±0.12	11.26±2.62**	1.92±0.36	10.23±3.22*

Figure5a	Denerv. 28d		Denerv. 28d	
	Ctrl Inn	Ctrl Den	TSCmKO Inn	TSCmKO Den
HDAC4^{P632}/HDAC4	1.00±0.22	2.43±0.08**	1.49±0.37	1.17±0.18 $$
HDAC4^{P632}/Actinin	1.00±0.13	17.33±2.13****	1.63±0.23	8.58±1.73**$$
HDAC4^{P246}/HDAC4	1.00±0.15	3.21±0.67*	1.93±0.54	4.69±0.60**
HDAC4^{P246}/Actinin	1.00±0.05	22.50±5.71**	2.18±0.57	33.67±4.99***
HDAC4 pan	1.00±0.08	6.87±1.01***	1.23±0.39	7.01±1.06***

Figure S5b	Denerv. 3d - Sol		Denerv. 3d - Sol		Denerv. 28d - Sol		Denerv. 28d - Sol	
	Ctrl Inn	Ctrl Den	TSCmKO Inn	TSCmKO Den	Ctrl Inn	Ctrl Den	TSCmKO Inn	TSCmKO Den
HDAC4^{P632}/HDAC4	1.00±0.27	2.06±0.76	1.92±0.45	2.71±0.70	1.00±0.27	0.26±0.05*	0.57±0.18	0.11±0.05
HDAC4^{P632}/Actinin	1.00±0.31	3.25±0.47*	1.57±0.1	4.96±0.98**	1.00±0.08	4.54±0.71*	1.05±0.30	3.66±1.52$^{0.06a}$
HDAC4^{P246}/HDAC4	1.00±0.36	1.62±0.54	1.08±0.08	0.84±0.34	1.00±0.11	0.17±0.02****	0.84±0.06	0.23±0.09***
HDAC4^{P246}/Actinin	1.00±0.48	2.27±0.36	0.92±0.22	1.35±0.56	1.00±0.11	2.85±0.26	1.45±0.03	7.01±2.69*
HDAC4	1.00±0.20	8.55±0.44*	2.65±0.22	11.56±2.98**	1.00±0.17	16.70±3.17**	1.69±0.10	32.49±3.40****$$

Figure S5g	Denerv. 1d		Denerv. 1d		Denerv. 3d		Denerv. 3d	
	Ctrl Inn	Ctrl Den	TSCmKO Inn	TSCmKO Den	Ctrl Inn	Ctrl Den	TSCmKO Inn	TSCmKO Den
CaMKIIβM^{P287}/CaMKIIβM	1.00±0.15	1.07±0.45	0.93±0.19	0.84±0.28	1.00±0.15	1.49±0.49	0.49±0.23	0.93±0.27
CaMKIIβM^{P287}/actinin	1.00±0.18	0.80±0.11	0.26±0.03$$	0.28±0.05$	1.00±0.16	1.75±0.48	0.23±0.05	0.46±0.01$
CaMKIIbM	1.00±0.18	1.15±0.21	0.35±0.05$	0.44±0.07$	1.00±0.08	1.25±0.22	0.59±0.17	0.57±0.13$

Figure S5g	Denerv. 7d		Denerv. 7d		Denerv. 28d		Denerv. 28d	
	Ctrl Inn	Ctrl Den	TSCmKO Inn	TSCmKO Den	Ctrl Inn	Ctrl Den	TSCmKO Inn	TSCmKO Den
CaMKIIβM^{P287}/CaMKIIβM	1.00±0.05	1.87±0.13**	0.61±0.19	1.36±0.29*	1.00±0.12	0.97±0.11	1.40±0.32	1.32±0.09
CaMKIIβM^{P287}/actinin	1.00±0.17	1.24±0.06	0.19±0.04$$$	0.52±0.05$$$	1.00±0.11	2.00±0.32	0.43±0.05	1.58±0.60*
CaMKIIβM	1.00±0.15	0.68±0.06*	0.34±0.04$$	0.41±0.06	1.00±0.10	2.15±0.57	0.34±0.09	1.23±0.52

Figure 6d	Innerv.				Denerv. 3d			
	Ctrl	iTSC untr.	iTSC Ig	iTSCsh	Ctrl	iTSC untr.	iTSC Ig	iTSCsh
HDAC4	1.00±0.30	1.22±0.05	0.83±0.06	0.87±0.29	1.00±0.08	1.03±0.15	0.88±0.15	0.66±0.12

Figure 8b	Denerv. 3d		Denerv. 3d		Denerv. 3d	
	Ctrl Inn	Ctrl Den	Akt1-TG Inn	Akt1-TG Den	TSCmKO Inn	TSCmKO Den
Akt^{P473}/Akt	1.00±0.35	0.56±0.04	0.25±0.07$$	0.20±0.02	0.40±0.04$	0.16±0.03
Akt^{P473}/ actinin	1.00±0.26	1.21±0.14	0.33±0.05$$	0.42±0.03$$	0.63±0.07	0.52±0.18$
Akt	1.00±0.15	2.02±0.12**	1.37±0.17	1.99±0.08*	1.45±0.03	2.87±0.56**$
S6$^{P235/6}$/S6	1.00±0.46	3.44±0.06*	3.07±0.17$^{0.06b}$	5.31±1.51$^{0.05a}$	4.01±0.28$	4.18±0.03
S6$^{P235/6}$/ actinin	1.00±0.30	3.10±0.49**	2.75±0.25$	3.79±0.69	3.61±0.49$$	5.24±0.99$
S6	1.00±0.30	0.78±0.06	0.81±0.11	0.67±0.06	0.80±0.05	1.13±0.22

Figure 8f	Denerv. 3d		Denerv. 3d	
	Ctrl Inn	Ctrl Den	Akt1-TG Inn	Akt1-TG Den
HDAC4^{P632}/HDAC4	1.00±0.02	1.71±0.41	1.09±0.14	4.60±0.71***$$
HDAC4^{P632}/ actinin	1.00±0.08	13.64±2.35	4.60±0.69	47.54±9.49***$$
HDAC4^{P246}/HDAC4	1.00±0.12	0.72±0.10	0.24±0.05$$	0.87±0.23*
HDAC4^{P246}/ actinin	1.00±0.16	5.72±0.40*	0.95±0.09	9.10±2.60**
HDAC4	1.00±0.05	8.44±1.40***	4.30±0.56$	10.36±1.23**

Values are mean±s.e.m.; n numbers are indicated in each figure legends. Absolute values were normalized on the corresponding total protein or on actinin (when unspecified), and on control

innervated muscle or muscle from untreated muscle. Significance between two conditions was assessed using two-tailed unpaired Student's *t*-test or one-/two-way ANOVA; * p<0.05; **p<0.01; ***p<0.001; **** p<0.0001 or a p value indicated with "a" when comparing innervated *vs.* denervated; $^{\$}$ p<0.05; $^{\$\$}$ p<0.01; $^{\$\$\$}$ p<0.001; $^{\$\$\$\$}$ p<0.0001 or a p value indicated with "b" when comparing genotypes or treatments.

Supplementary Table 2 List of primers

Gene	Forward sequence	Reverse sequence
Akt1	actcattccagacccacgac	gtccagggcagacacaatct
Akt2	tggttatgggggtcatctgt	catgctactctggcactcca
Akt3	gaaactggccacttctgctc	actgaggtgtggtggagacc
Camk2b	tgaagacatcgtggcaagag	aggcttgaggtctctgtgga
Camk2d	ctggcacacctgggtatctt	atcccagaagggtgggtatc
Camk2g	tcgccaagagacggtagagt	ttggcagctgaaaagttcct
Ctsl	gtggactgttctcacgctca	tccgtccttcgcttcatagg
Chrna1	tcccttcgatgagcagaact	gggcagcaggagtagaacac
Chrng	gccctggagtcagctatgag	ggtaaaaccttgcccagtca
Dach2	ccagctcaaatcccagtcat	cgcagttccttcttttcctg
Eno3	gggagatgacctcacggtaa	ttacaggcctggatggactc
Fbxo32	caacattaacatgtgggtgtat	gtcactcagcctctgcatg
Gabarapl1	catcgtggagaaggctccta	atacagctggcccatggtag
Gadd45	gggctcagagatgactttgc	tttttgtcccttttgccttg
Gfp	agaacggcatcaaggtgaac	tgctcaggtagtggttgtcg
Hdac4	cagacagcaagccctcctac	agacctgtggtgaaccttgg
Myh2	acaaatctatccaagttccg	ttcggtcattccacagcatc
Myh4	cagatgaaaaggtggccatt	cttccctttgcttttgcttg
Myod1	agtgaatgaggccttcgaga	caggatctccaccttgggta
Myog	cactcccttacgtccatcgt	cagggctgttttctggacat
Pfkm	gatgcaaggacttccgagag	gctccactctgaacggaaag
Mitr	ccaccttgaagaagcagagg	tggtgtccttagaggctgct
Mtor	aaacacttcggagagctgga	cgggtcttccttgtttgtgt
Rptor	ggtacaagcagagcctcgac	gacccagacctctccattca
Rps6	gtatgcacccgcttaggtgt	aacacggtggagaaaacctg
Tbp	ctcagttacaggtggcagca	cagcacagagcaagcaactc
Trim63	acctgctggtggaaaacatc	aggagcaagtaggcacctca
