# Supplementary material for: mTORC1 and PKB/Akt control the muscle response to denervation by regulating autophagy and HDAC4
Source: Nat Commun. 2019 Jul 18;10:3187. doi: 10.1038/s41467-019-11227-4 (PMC6639401; doi:10.1038/s41467-019-11227-4)
Supplement: Supplementary file 2 — Reporting Summary [file 41467_2019_11227_MOESM2_ESM.pdf]

nature research

Corresponding author(s): Perrine Castets
Markus Rüegg

Last updated by author(s): Jun 3, 2019

Reporting Summary

Nature Research wishes to improve the reproducibility of the work that we publish. This form provides structure for consistency and transparency in reporting. For further information on Nature Research policies, see Authors & Referees and the Editorial Policy Checklist.

Statistics

For all statistical analyses, confirm that the following items are present in the figure legend, table legend, main text, or Methods section.

n/a	Confirmed	
☐	☒	The exact sample size (n) for each experimental group/condition, given as a discrete number and unit of measurement
☐	☒	A statement on whether measurements were taken from distinct samples or whether the same sample was measured repeatedly
☐	☒	The statistical test(s) used AND whether they are one- or two-sided<br>*Only common tests should be described solely by name; describe more complex techniques in the Methods section.*
☐	☒	A description of all covariates tested
☐	☒	A description of any assumptions or corrections, such as tests of normality and adjustment for multiple comparisons
☐	☒	A full description of the statistical parameters including central tendency (e.g. means) or other basic estimates (e.g. regression coefficient) AND variation (e.g. standard deviation) or associated estimates of uncertainty (e.g. confidence intervals)
☒	☐	For null hypothesis testing, the test statistic (e.g. F, t, r) with confidence intervals, effect sizes, degrees of freedom and P value noted<br>*Give P values as exact values whenever suitable.*
☒	☐	For Bayesian analysis, information on the choice of priors and Markov chain Monte Carlo settings
☒	☐	For hierarchical and complex designs, identification of the appropriate level for tests and full reporting of outcomes
☒	☐	Estimates of effect sizes (e.g. Cohen's d, Pearson's r), indicating how they were calculated

Our web collection on statistics for biologists contains articles on many of the points above.

Software and code

Policy information about availability of computer code

Data collection	StepOne software v2.3 was used for qPCR data collection<br>AnalySIS software (Soft Imaging System) was used for image acquisition<br>Leica LAS AF Software was used for confocal acquisition
Data analysis	StepOne software was used for qPCR data collection<br>ImageJ-win64 was used for protein quantification<br>AnalySIS software (Soft Imaging System) was used for histology analysis<br>GraphPad Prism 7 Software was used for statistical analysis<br>Imaris software was used for GFP-LC3 quantification<br>Fiji and Matlab software were used for turnover analysis

For manuscripts utilizing custom algorithms or software that are central to the research but not yet described in published literature, software must be made available to editors/reviewers. We strongly encourage code deposition in a community repository (e.g. GitHub). See the Nature Research guidelines for submitting code & software for further information.

Data

Policy information about availability of data

All manuscripts must include a data availability statement. This statement should provide the following information, where applicable:

- Accession codes, unique identifiers, or web links for publicly available datasets
- A list of figures that have associated raw data
- A description of any restrictions on data availability

The data presented in this study are available from the corresponding authors upon reasonable request.

Field-specific reporting

Please select the one below that is the best fit for your research. If you are not sure, read the appropriate sections before making your selection.

☒ Life sciences ☐ Behavioural & social sciences ☐ Ecological, evolutionary & environmental sciences

For a reference copy of the document with all sections, see nature.com/documents/nr-reporting-summary-flat.pdf

Life sciences study design

All studies must disclose on these points even when the disclosure is negative.

Sample size	Experiments were conducted on 3 or more independent biological samples. Sample sizes were empirically determined based on prior experience in the lab regarding muscle analysis.
Data exclusions	No exclusion
Replication	In vivo results were reproducible accross at least 3 independent animals and experiments. In vitro procedures were repeated at least 3 times, with reproducible results.
Randomization	Animals were randomly allocated to experiments, using littermate and sex-matched control animals.
Blinding	For visual quantification (GFP-LC3 counts, fragmentation, ...), the investigators were blinded to group allocation during data analysis.

Reporting for specific materials, systems and methods

We require information from authors about some types of materials, experimental systems and methods used in many studies. Here, indicate whether each material, system or method listed is relevant to your study. If you are not sure if a list item applies to your research, read the appropriate section before selecting a response.

Materials & experimental systems

n/a	Involved in the study	
☐	☒	Antibodies
☐	☒	Eukaryotic cell lines
☒	☐	Palaeontology
☐	☒	Animals and other organisms
☒	☐	Human research participants
☒	☐	Clinical data

Methods

n/a	Involved in the study	
☒	☐	ChIP-seq
☒	☐	Flow cytometry
☒	☐	MRI-based neuroimaging

Antibodies

Antibodies used	The following antibodies were used: PKB/Akt (#9272), Phospho-AktSer473 (#9271), Phospho-AktSer308 (#9275), p70 S6 kinase (#9202), Phospho-p70 S6 kinaseThr389 (#9205), S6 Ribosomal Protein (#2217), Phospho-S6 Ribosomal ProteinSer235/6 (#2211), Phospho-S6 Ribosomal ProteinSer240 (#2215), LC3B (#2775), Ulk1 (#8054), Phospho-Ulk1Ser757 (#6888), Phospho-Ulk1Ser317 (#6887) Beclin1 (#3495), HDAC4 (#15164 and #7628), Phospho-HDAC4Ser246 (#3443), Phospho-HDAC4Ser632 (#3424), nucleolin (#14574), endonuclease G (#4969), Gadd45 (#4632), Rab5 (#2143), Rab7 (#9367) from Cell Signaling; α-actinin (A5044) and Neurofilament 200 (N4142) from Sigma; p62 (GP62C) from Progen; Myogenin (F5D), Myosin Heavy Chain types I (A4.840), IIA/IIX (A4.74), IIB (BF-F3) from the Developmental Studies Hybridoma Bank; Laminin (ab11575 and ab11576) from Abcam; Lamin B from Santa Cruz (C-20); Synaptophysin (A0010) from Dako; acetylated Histones H3 (17-658) and H4 (06-598), Trimethyl Histone H3 (Lys4 - 17-614) from Millipore Merck.
Validation	All primary antibodies were commercially available and validated by the distributors. Correct molecular weight and/or localization was confirmed during the study.

Eukaryotic cell lines

Policy information about cell lines

Cell line source(s)	C2C12 myoblast/myotubes from ATCC
Authentication	Cells were authenticated by confirming their fusion in myotubes upon differentiation.
Mycoplasma contamination	Cells were not tested for mycoplasma contamination.

Commonly misidentified lines (See ICLAC register)	None

Animals and other organisms

Policy information about studies involving animals; ARRIVE guidelines recommended for reporting animal research

Laboratory animals	C57Bl6/J mice - Strains: TSCmKO, RAmKO, Akt1-TG, inducible TSCmKO, GFP-LC3 mice<br>All mice were 8 to 12 weeks of age at the denervation time. Both male and female were used.
Wild animals	No wild animals were used in this study.
Field-collected samples	No field-collected samples were used in this study.
Ethics oversight	All animal studies were performed in accordance with the European Union guidelines for animal care and approved by the Swiss authorities.

Note that full information on the approval of the study protocol must also be provided in the manuscript.
